# Role of electrocardiographic early repolarization pattern in long-term outcomes of a community-based middle-aged and geriatric ambulatory population: a prospective cohort study

**DOI:** 10.18632/aging.202369

**Published:** 2020-12-19

**Authors:** Jyh-Ming Jimmy Juang, Yu-Jyun Huang, I-Shou Chang, Ching-Yu Julius Chen, I-Chien Wu, Chih-Cheng Hsu, Tzu-Yu Chen, Wei-Ting Tseng, Shih-Fan Sherri Yeh, Chao Agnes Hsiung

**Affiliations:** 1 Cardiovascular Center and Division of Cardiology, Department of Internal Medicine, National Taiwan University Hospital and National Taiwan University College of Medicine, Taipei City, Taiwan; 2 Division of Biostatistics, Institute of Epidemiology and Preventive Medicine, National Taiwan University, Taipei City, Taiwan; 3 Institute of Population Health Sciences, National Health Research Institutes, Zhunan, Taiwan; 4 Department of Environmental and Occupational Medicine, National Taiwan University Hospital Hsin-Chu Branch, Hsin-Chu, Taiwan

**Keywords:** prevalence, long-term prognosis, early repolarization pattern, Han Chinese population, community-based

## Abstract

In some studies, electrocardiographic early repolarization pattern (ERP) has been associated with an increased risk of death from cardiac causes. However, little is known about the prognostic significance of ERP in the middle-aged and geriatric general populations. We investigated the prevalence and long-term prognostic significance of early repolarization pattern (ERP) on electrocardiograms (ECGs) in the Healthy Aging Longitudinal Study (HALST) cohort of 4615 middle-aged and geriatric community-dwelling Han Chinese adults from Taiwan. The study subjects were followed-up for 95±22 months. A positive ERP of ≥0.1 mV was observed in 889 (19.3%) of the subjects. Kaplan-Meier survival curve analysis showed that ERP was not associated with all-cause and cardiovascular mortality (log-rank test, *P*=0.13 and 0.84, respectively). Cox regression analysis after adjusting for covariables revealed that age, blood pressure, smoking, diabetes, stroke, chronic kidney disease, and corrected QT interval (QTc) were associated with increased risk of all-cause mortality (*P*<0.05). Age, and stroke were risk factors associated with increased risk of cardiovascular mortality (*P*<0.05). However, ERP alone was not associated with all-cause or cardiovascular mortality. These findings show that ERP is common in the middle-aged and geriatric Han-Chinese individuals from the HALST cohort and is not associated with all-cause or cardiovascular mortality.

## INTRODUCTION

Sudden cardiac death (SCD) is a major health issue worldwide and accounts for 180,000 to 250,000 deaths annually in the United States [1]. The age-adjusted incidence of SCD in the United States is 60 per 100,000 population [2]. Ventricular tachyarrhythmia is the major cause of SCD, and is not associated with any structural heart disease in 6 to 14% of cases [3, 4]. In some cases, SCD is associated with electrocardiographic abnormalities that affect ventricular repolarization, such as long or short QT syndrome [5]. Early repolarization pattern (ERP) is characterized by elevation of the QRS-ST junction or the J-point in a surface 12-lead electrocardiogram (ECG). Although ERP was considered benign, recent studies have suggested its potential association with cardiac arrhythmogenicity [6]. In case-control studies, the presence of ERP in the inferior or lateral leads is associated with susceptibility to ventricular fibrillation and SCD in patients without structural heart disease [7–9].

ERP is a common electrocardiographic finding that affects 1% to 13% of adults and is more common in young athletic males [7, 9–12]. The age of individuals enrolled in previous studies regarding ERP ranged widely from 25 to 95 years [13, 14]. Sinner et al conducted a large, prospective, population-based case-cohort study of individuals of Central-European descent (MONICA/KORA) and reported a high prevalence of ERP (13.1%) in individuals aged between 35-74 years and a 2- to 4-fold increased risk of cardiovascular mortality in individuals with ERP and aged between 35-54 years [15]. Haruta et al reviewed ECG records of 5976 atomic bomb survivors in Nagasaki, Japan with a mean age of 47.2 years and reported that ERP was associated with elevated risk of unexpected death and decreased risk of cardiac and all-cause death [16]. However, the long-term prognostic significance of ERP is poorly characterized in older middle-aged and elderly population.

In this study, we investigated the prevalence and prognostic value of ERP regarding cardiac and all-cause mortality in a large, multi-site, Healthy Aging Longitudinal Study (HALST) cohort consisting of older middle-aged and elderly adults belonging to the Han Chinese population in Taiwan.

## RESULTS

### Study participants

**Figure 1:**
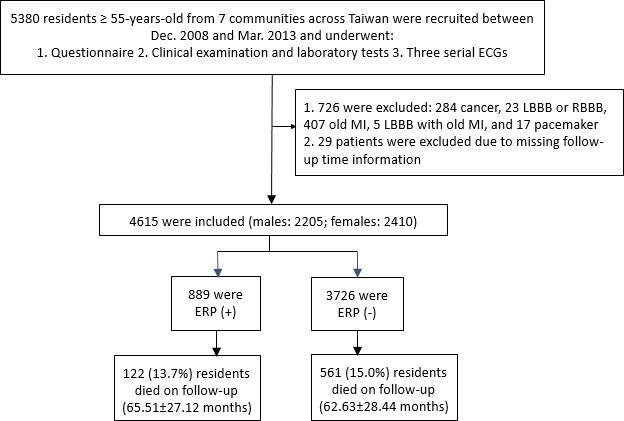
**Schematic representation of the enrollment and inclusion criteria for the study subjects.** ECG, electrocardiogram; LBBB, left bundle branch block; RBBB, right bundle branch block; MI, myocardial infarction; ERP, early repolarization pattern

The flowchart of enrollment and inclusion criteria of the study subjects is shown in Figure 1. We initially recruited 5,380 relatively healthy and ambulatory individuals from 7 communities across Taiwan. We then excluded 755 individuals with cancer and underlying severe cardiovascular diseases (e.g., myocardial infarction or pacemaker implantation), as well as those with missing follow-up information. Finally, we included 4,615 healthy individuals in this study. The prevalence of ERP in the study cohort was 19.3% (n=889/4,615). In the ERP (+) group, 122 out of 889 individuals died during follow-up (65.51±27.12 months). In the ERP (-) group, 561 out of 3726 individuals died during follow-up (62.63±28.44 months).

The baseline clinical and demographic data of the study population is shown in Table 1. The mean age was 69.1±8.2 years and 47.8% of the study subjects were males. The average age of study participants was lower and the proportion of males and current smokers was higher in the ERP (+) group compared to the ERP (-) group. The mean values for the body mass index (BMI), systolic blood pressure (SBP), diastolic blood pressure (DBP), and hypertension were significantly lower (all *P*<0.005) and the length of the corrected QT interval (QTc) was shorter (*P*<0.001) in the ERP (+) group compared to the ERP (-) group.

**Table 1:** Demographic and clinical characteristics of the study population*.

**Parameters**	**All (N=4615)**	**ERP(+) (n=889)**	**ERP(-) (n=3,726)**	***P*-value**
Male, n (%)	2205 (47.8%)	470 (52.9%)	1735 (46.6%)	<0.001
Age at enrollment (years)	69.1±8.2	67.7±7.8	69.5±8.2	<0.001
BMI (kg/m^2^)	24.5±3.5	24.1±3.3	24.6±3.5	<0.001
Systolic BP (mmHg)	128.4±18.7	125.89±18.7	128.9±18.7	<0.001
Diastolic BP (mmHg)	70.5±10.7	69.4±10.5	70.7±10.7	<0.001
Smoking				<0.001
Current	608 (13.2%)	168 (18.9%)	440 (11.8%)	
Quit	716 (15.5%)	132 (14.8%)	584 (15.8%)	
Never	3291 (71.3%)	589 (66.3%)	2702 (72.5%)	
Essential hypertension, n (%)	2011 (43.6%)	349 (39.3%)	1662 (44.6%)	0.004
Diabetes mellitus, n (%)	823 (17.8%)	159 (17.9%)	664 (17.8%)	0.99
Stroke, n (%)	231 (5%)	36 (4%)	195 (5%)	0.171
Hyperlipidemia, n (%)	1456 (31.5%)	269 (30.2%)	1187 (31.8%)	0.378
CKD, n (%)	667 (14.6%)	126 (14.1%)	541 (14.5%)	0.833
PR interval, *ms*	171.1±34.1	170.7±32.5	171.2±34.4	0.677
QTc, ms	437.0±22.6	431.4±20.7	438.3±22.9	<0.001

ERP: early repolarization pattern by the criteria adopted in 2015 [35]; BMI: body mass index; BP: blood pressure; CKD: chronic kidney disease; *Values are presented as n (%) or mean ± standard deviation; QTc was calculated by Bazett’s equations.

**Figure 2:**
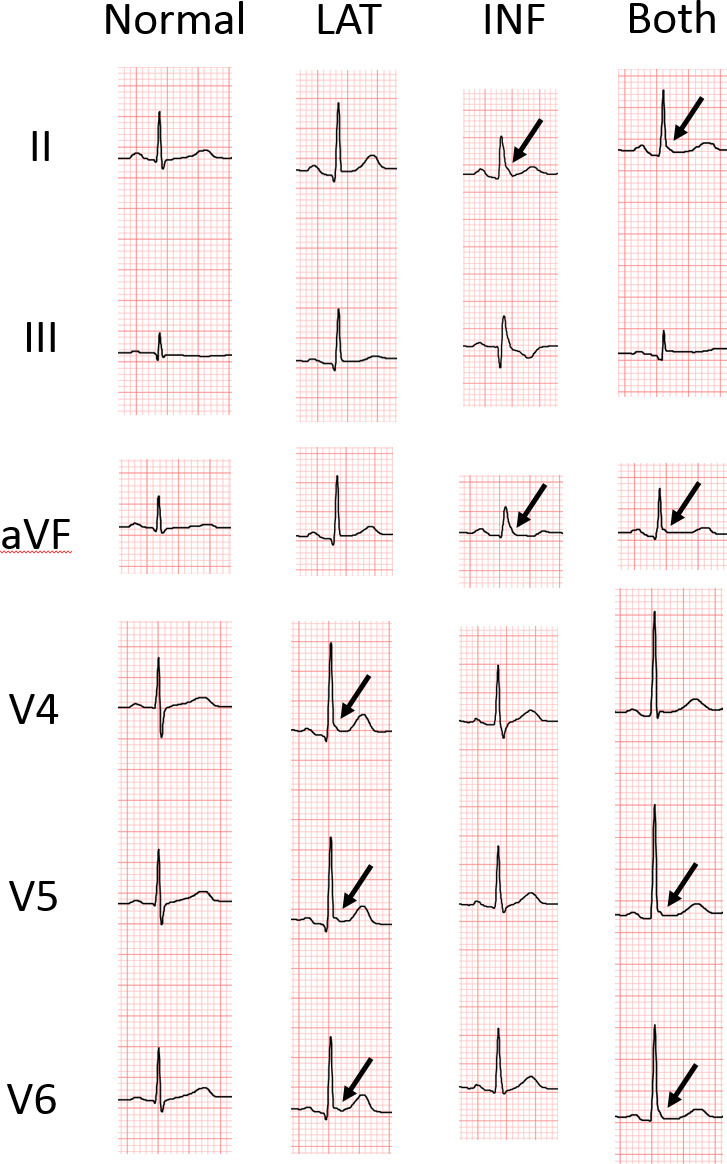
**Representative ECGs of individuals with and without early repolarization pattern (ERP).** LAT, ERP in lateral leads; INF, ERP in inferior leads; Both, ERP in both inferior and lateral leads. The arrow indicates junction (J)-point elevation greater than 0.1 mV.

Figure 2 shows the representative ECGs of few selected individuals belonging to ERP (+) and ERP (-) groups. ERP was observed in the inferior and lateral leads, both separately and simultaneously. Among the 4,615 healthy individuals, 574 (12.4%) individuals were ERP-positive in the inferior leads (ERP-inf^+^), 214 (4.6%) individuals were ERP-positive in the lateral leads (ERP-lat^+^), and 101(2.2%) individuals were ERP-positive in both lateral and inferior leads (ERP-lat^+^ inf^+^). The agreement between the two initial interpreters was moderate to high (k=0.97, agreement proportion= 0.98).

### Association between positive ERP and all-cause and cardiovascular mortality

**Figure 3:**
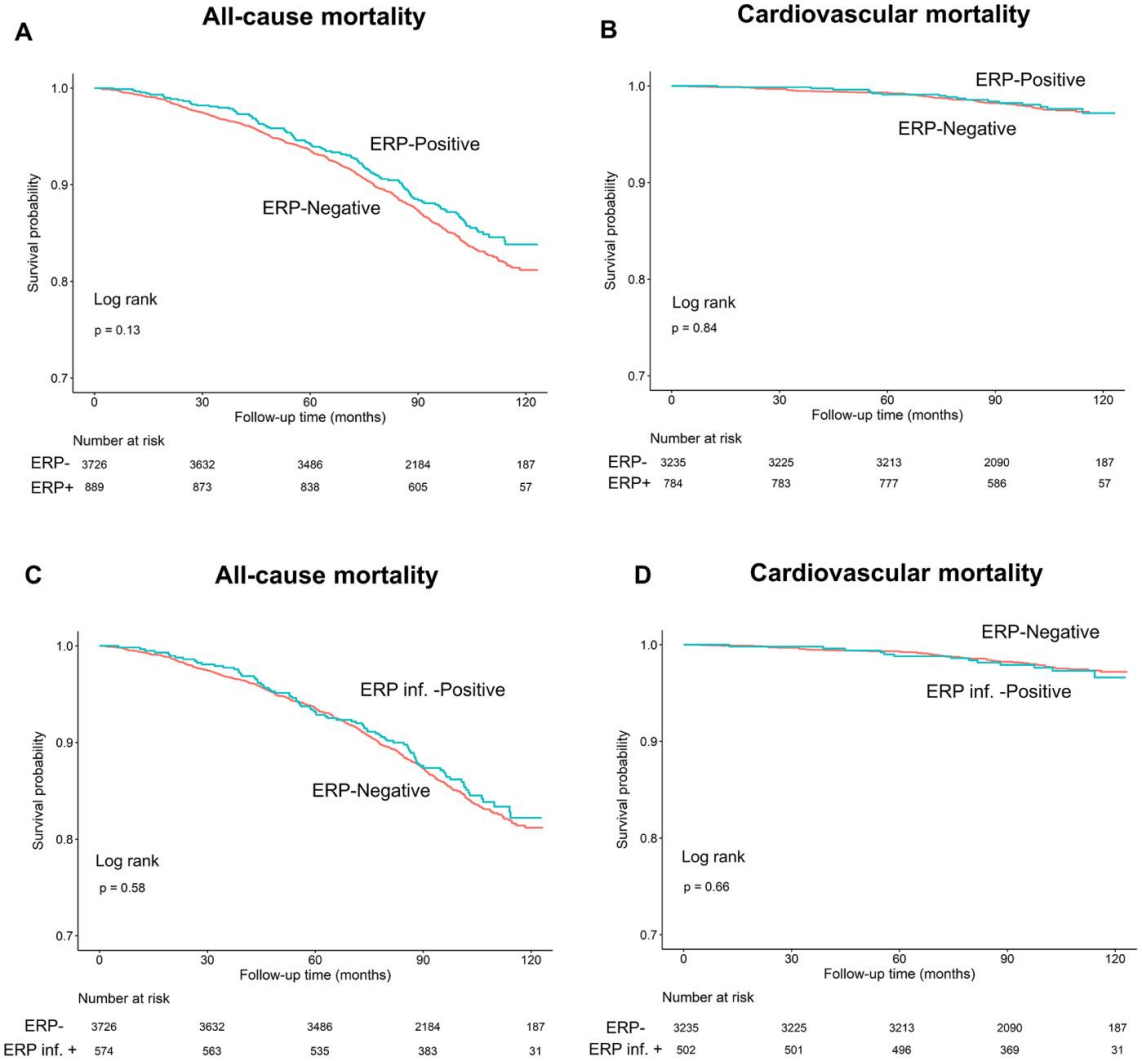
****Kaplan–Meier survival curves show (**A**, **C**) all-cause and (**B**, **D**) cardiovascular mortality rates in individuals with and without early repolarization pattern, and individuals with and without early repolarization pattern in inferior leads.

We observed that all-cause and cardiovascular mortality rates were similar in ERP (+) and ERP (-) groups during a mean follow-up time of 95.1±21.9 months (log-rank test, *P*=0.13 and *P*=0.84, respectively; Figure 3A, 3B). Since previous studies showed that ERP in the inferior leads was a risk factor for all-cause and cardiovascular mortality [17], we performed Kaplan-Meier survival curve analysis to investigate the association between the two types of mortality and positive ERP in the inferior leads (ERP-inf^+^). The results showed that all-cause and cardiovascular mortality rates were similar for individuals with and without ERP in the inferior leads (log-rank test, *P*=0.58, 0.66, Figure 3C, 3D).

### Long-term outcomes of all-cause mortality and cardiovascular mortality and ERP stratified by age

**Figure 4:**
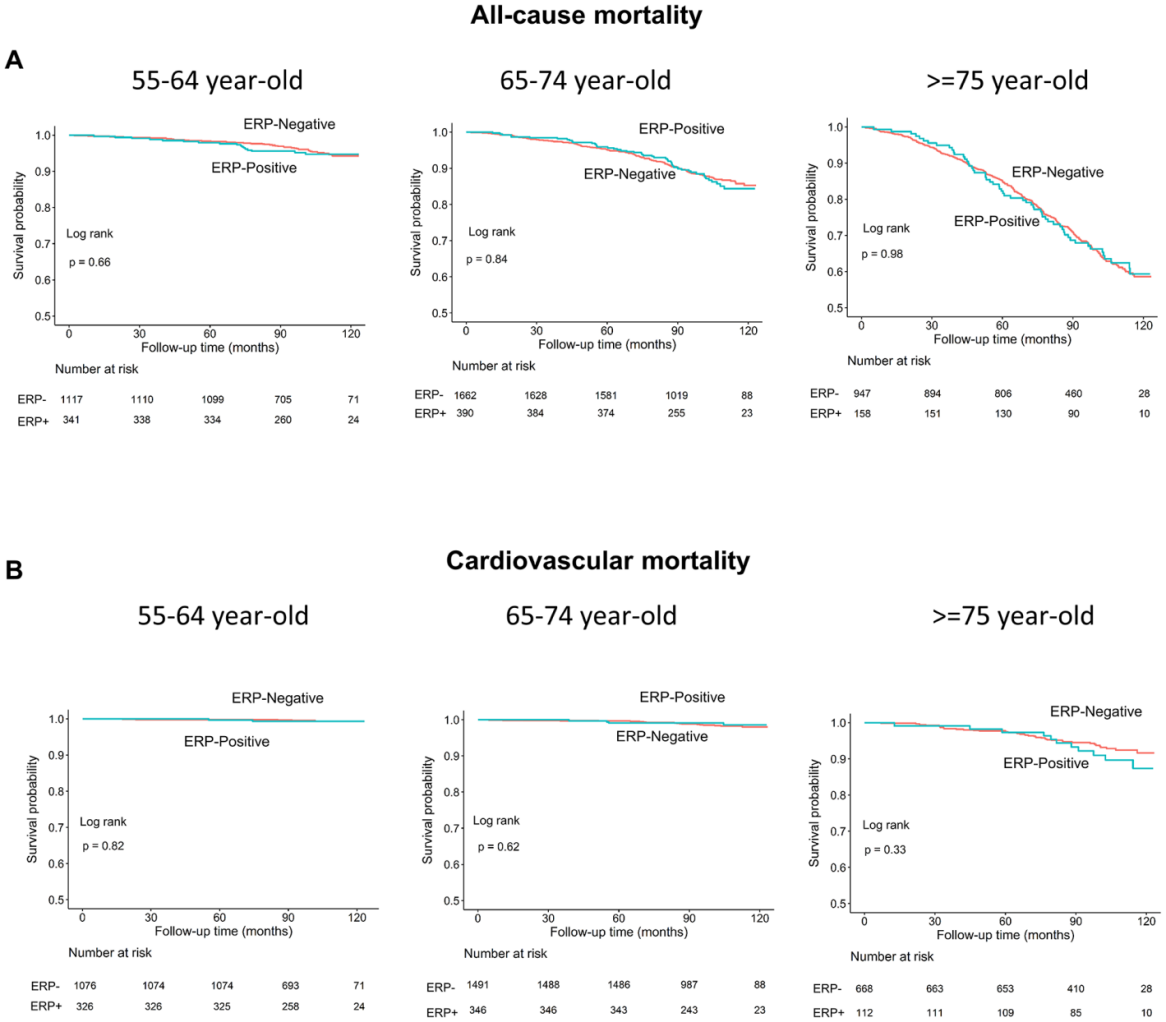
****Kaplan–Meier survival curves show (**A**) all-cause and (**B**) cardiovascular mortality rates of individuals with and without early repolarization pattern stratified by age.

**Figure 5:**
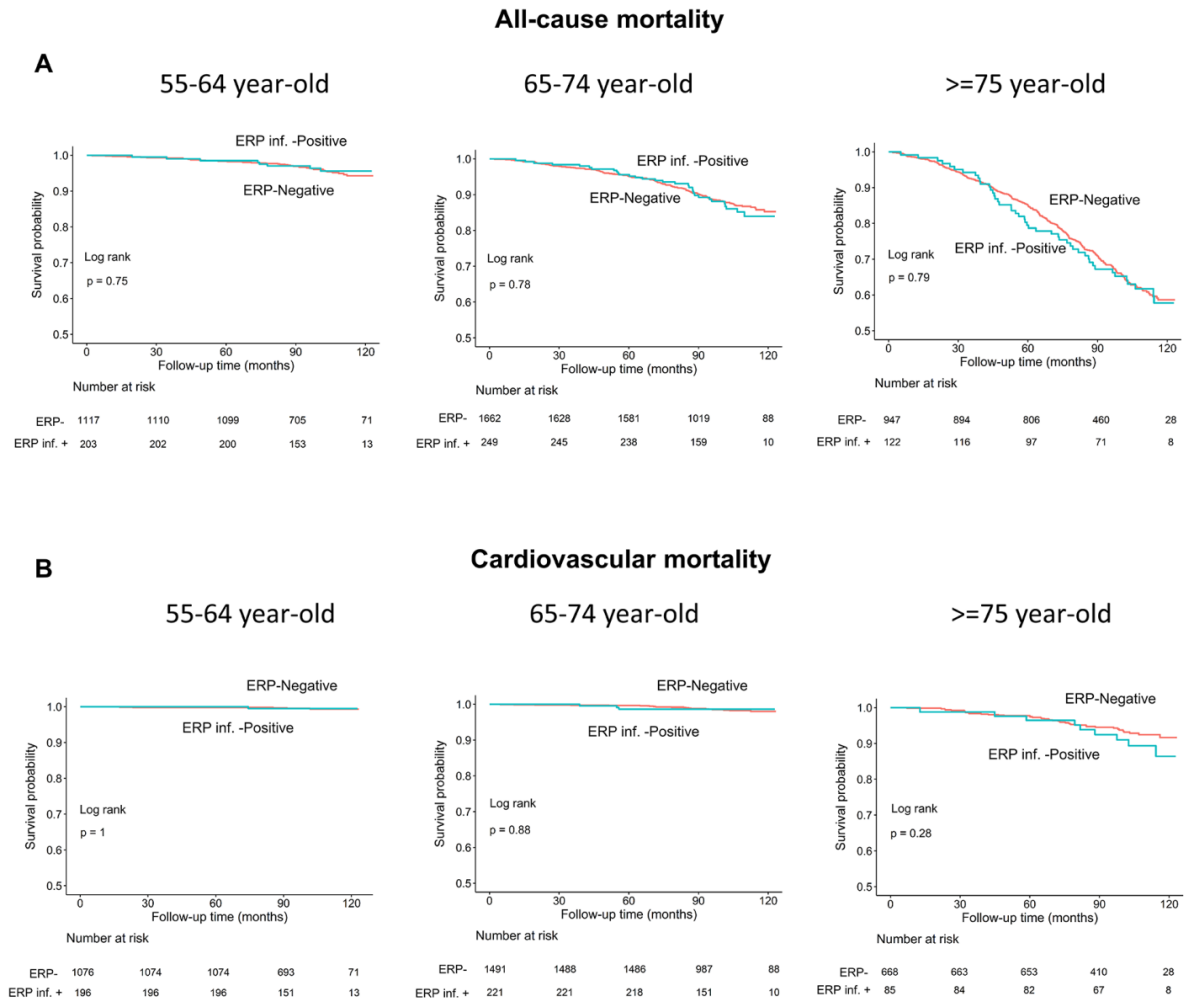
Kaplan–Meier survival curves show (**A**) all-cause and (**B**) cardiovascular mortality rates of individuals with and without early repolarization pattern in inferior leads stratified by age.

Age is a known risk factor for both all-cause and cardiovascular mortality [18]. Therefore, we performed subgroup analyses in 3 age groups (55-64, 65-74, and ≥75 years) and observed no differences in all-cause or cardiovascular mortality rates in individuals with and without ERP in each subgroup (log-rank test, all *P*>0.05; Figure 4A, 4B). Moreover, the survival rates of individuals with and without ERP in the inferior leads among the 3 age subgroups were also similar (all *P*>0.05; Figure 5A, 5B).

### Correlation between ERP and other risk factors with all-cause and cardiovascular mortality

As shown in Tables 2, 3 and Supplementary Figures 1, 2, multivariate Cox proportional hazards analyses after adjusting for covariables showed that ERP was not a risk factor for both all-cause and cardiovascular disease mortality (*P*=0.12 and 0.7, respectively). Furthermore, our study showed that age, gender, BMI, SBP, DBP, smoking, diabetes mellitus, stroke, chronic kidney disease, and QTc were risk factors for all-cause mortality (all *P* values <0.05) and age and stroke were risk factors for cardiovascular mortality (all *P* values <0.05, Supplementary Figure 2). Similarly, after adjusting for covariables, multivariate analysis showed that individuals with ERP in inferior leads alone (ERP-inf ^+^) were not associated with increased risk of all-cause and cardiovascular mortality (both *P* values >0.05; Supplementary Figures 3, 4). Moreover, multivariate analysis showed that patients with ERP in lateral leads alone (ERP-lat ^+^) and ERP in both inferior leads and lateral leads (ERP-inf ^+^/lat ^+^) were associated with risk of all-cause mortality (*P*=0.029, 0.048*,* respectively; Supplementary Figures 5, 6), but were not associated with the risk of cardiovascular mortality (both *P* values >0.05; Supplementary Figures 7, 8) after adjusting for covariables. Although patients with ERP-inf ^+^/lat ^+^ were associated with increased risk of all-cause mortality, the data was not sufficient to make a strong conclusion because only 5 deaths were reported in this group during the 10-year follow-up.

**Table 2:** Association between ERP and all-cause mortality*.

		**Hazard ratio (95% CI)**	***P*-value**
All			
	ERP (reference: negative)	0.98 (0.81, 1.20)	0.877
	Gender (reference: male)	0.71 (0.57, 0.87)	0.001
	Age	1.11 (1.10, 1.12)	<0.001
	BMI (kg/m^2^)	0.96 (0.94, 0.98)	<0.001
	Systolic BP (mmHg)	1.01 (1.01, 1.02)	<0.001
	Diastolic BP (mmHg)	0.99 (0.98, 0.99)	0.003
	Smoking (reference: current)		
	Quit	0.63 (0.50, 0.79)	<0.001
	Never	0.50 (0.40, 0.63)	<0.001
	Diabetes mellitus	1.51 (1.26, 1.81)	<0.001
	Stroke	1.94 (1.52, 2.48)	<0.001
	Chronic kidney disease	1.35 (1.10, 1.66)	0.005
	QTc	1.01 (1.00, 1.01)	<0.001
Age-stratified			
55-64 y			
	ERP (reference: negative)	1.04 (0.58, 1.86)	0.886
	Gender (reference: male)	0.34 (0.16, 0.73)	0.006
	Smoking (reference: current)		
	Quit	0.63 (0.31, 1.27)	0.198
	Never	0.40 (0.21, 0.79)	0.008
	QTc	1.01 (1.00, 1.03)	0.012
65-74 y			
	ERP (reference: negative)	1.01 (0.73, 1.39)	0.953
	Gender (reference: Male)	0.56 (0.39, 0.80)	0.001
	Systolic BP (mmHg)	1.02 (1.00, 1.03)	0.008
	Diastolic BP (mmHg)	0.97 (0.96, 0.99)	0.004
	Smoking (reference: current)		
	Quit	0.66 (0.45, 0.97)	0.032
	Never	0.44 (0.31, 0.64)	<0.001
	Stroke	1.98 (1.33, 2.95)	0.001
	QTc	1.01 (1.00, 1.01)	0.001
≥75 y			
	ERP (reference: negative)	0.87 (0.65, 1.17)	0.357
	BMI (kg/m^2^)	0.92 (0.89, 0.95)	<0.001
	Systolic BP (mmHg)	1.02 (1.01, 1.03)	<0.001
	Diastolic BP (mmHg)	0.98 (0.97, 0.99)	0.002
	Smoking (reference: current)		
	Quit	0.78 (0.55, 1.09)	0.150
	Never	0.68 (0.48, 0.96)	0.030
	Diabetes mellitus	1.42 (1.11, 1.81)	0.005
	Stroke	1.89 (1.35, 2.64)	<0.001
	QTc	1.01 (1.00, 1.01)	0.008

ERP: early repolarization pattern; BMI: body mass index; BP: blood pressure; CI, confidence interval; *Except for ERP, only significant variables (*P*<0.05) are shown here; QTc was calculated by Bazett’s equations.

**Table 3:** Association between ERP and cardiovascular mortality*.

		**Hazard ratio (95% CI)**	***P*-value**
All			
	ERP (reference: negative)	1.27 (0.74, 2.20)	0.386
	Age	1.13 (1.10, 1.17)	<0.001
	Stroke	2.11 (1.01, 4.42)	0.048
Age-stratified			
55-64 y			
	ERP (reference: negative)	1.08 (0.21, 5.68)	0.928
	BMI	1.23 (1.02, 1.47)	0.027
	CKD	4.83 (1.03, 22.61)	0.046
65-74 y			
	ERP (reference: negative)	0.83 (0.28, 2.47)	0.744
	Stroke	3.59 (1.30, 9.92)	0.014
	QTc	1.02 (1.00, 1.04)	0.017
≥75 y			
	ERP (reference: negative)	1.54 (0.77, 3.07)	0.219
	Systolic BP (mmHg)	1.03 (1.01, 1.05)	0.014

ERP: early repolarization pattern; BMI: body mass index; BP: blood pressure; CKD: chronic kidney disease; *Except for ERP, only significant variables (*P*<0.05) are shown here. QTc was calculated by Bazett’s equations.

Since age is a strong predictor of both all-cause and cardiovascular mortality [18], we further compared mortality rates by stratifying ERP (+) and ERP (-) individuals into 3 age subgroups (55-64, 65-74, and ≥75 years). Multivariate Cox proportional hazard analyses after adjusting for covariables showed that ERP was not a risk factor for both all-cause and cardiovascular mortality in all the age subgroups (all *P* values >0.05; Tables 2, 3, Supplementary Figures 9–14). Moreover, study subjects with ERP in inferior leads alone (ERP-inf ^+^), lateral leads alone (ERP-lat ^+^), or both inferior and lateral leads (ERP-inf ^+^/lat ^+^) were not associated with increased risk of both all-cause and cardiovascular mortality in all age subgroups (all *P* values >0.05; Supplementary Figure 15–32).

### Comparisons of the prevalence and clinical outcomes of ERP in community-based studies worldwide

As shown in Table 4, the prevalence of ERP ranged from ~1% to 25% in Caucasians and 3.5%-24% in Japanese according to the community based population studies worldwide [10, 13, 14, 16]. However, the association between all-cause and cardiovascular mortality and EPR was not consistent among the published studies. Most studies showed that ERP was not associated with all-cause mortality but was associated with cardiovascular mortality. Our study cohort included older middle-aged and geriatric individuals ≥55 years with an average age of 69.1±8.2 years (range: 55-103 years), which was the highest age average of a study cohort when compared with previous reports. Moreover, the prevalence of ERP was 19.26% in our study cohort. This was within the prevalence range reported by other studies (0.99-24.79%), but was higher than the mean worldwide prevalence of 6.42%. Overall, our study shows that ERP is not associated with increased risk of all-cause and cardiovascular mortality in older middle aged and geriatric Han-Chinese population in Taiwan.

**Table 4:** Summary of results from community-based studies worldwide regarding the prevalence of ERP, age distribution, and relationship with all-cause and cardiovascular mortality.

**Study**	**Year**	**Country**	**Screened population N**	**Male (%)**	**Age (years)**	**ERP (+)**			**All-cause mortality**	**Cardiovascular mortality**
						**N (%)**	**M (%)**	**F (%)**		
**North America**										
Klatsky et al.[10]	2003	USA	73,088	43.88	NA	670 (0.92)	583 (1.82)	87 (0.21)	Not increased	Increased
Olson et al.[19]	2011	USA	15,141	44.30	54.1	1866 (12.3)	1420 (21.17)	446 (5.29)	Not increased	SCD increased in whites and females
Uberoi et al.[40]	2011	USA	29,281	87.24	55±15	714 (2.44)	661 (2.59)	53 (1.42)	NA	Not increased
Perez et al.[41]	2012	USA	29,281	87	55	664 (2.27)	NA	NA	NA	Increased in non-African Americans but not in African Americans
Ilkhanoff et al.[13]	2014	USA	5,039	45.54	25 (18-30)	1249 (24.79)	1139 (49.63)	110 (4.01)	Not increased	Not increased
Pargaonkar et al.[42]	2015	USA	20,661	90.53	20-55	4219 (20)	3840 (20.53)	379 (19.38)	NA	Not increased
Leiderman et al.[43]	2019	USA	17,901	38.57	53±13	995 (5.6)	489 (7.08)	506 (4.60)	Not increased	NA
Europe										
Tikkanen et al.[25]	2009	Finland	10,864	52.40	44±8	630 (5.8)	407 (7.15)	223 (4.31)	NA	Increased
Sinner et al.[15]	2010	Germany	6,213	48.85	35-74	812 (13.1)	439 (14.46)	373 (11.74)	Increased	Increased
Rollin et al.[20]	2012	France	1,161	51.59	35-64	159 (13.7)	126 (21.04)	33 (5.87)	Increased	Increased
Asia										
Haruta et al.[16]	2011	Japan	5,676	43.71	47.2±15.4	779 (23.9)	815 (31.20)	614 (18.25)	Decreased	Decreased
Hisamatsu et al.[14]	2013	Japan	7,630	40.73	52.4 (30-95)	264 (3.5)	253 (8.14)	11 (0.24)	NA	Cardiac death and death from CAD increased
Juang et al.^present study^	2020	Taiwan	4,615	47.78	69.1±8.2 (55-103)	889 (19.26)	489 (7.08)	506 (4.60)	Not increased	Not increased
Total	-	-	226,851		-	14,560 (6.42)	10,642 (9.72)	3,254 (3.69)	-	-

ERP: early repolarization pattern; NA: not available, means no number was provided in the papers; USA: United States of America; M: male; F: female; SCD: sudden cardiac death; CAD: coronary artery disease

## DISCUSSION

The prevalence of cardiovascular disease is expected to increase because of a constant rise in the proportion of older individuals worldwide. ERP is not a rare event and has been reported in studies related to several ethnicities [19, 20], and has been associated with sudden cardiac arrest [15, 20]. To the best of our knowledge, this is the first study to examine the prevalence and prognostic value of ERP in a large cohort of older middle-aged and elderly individuals with a mean age ≥65 years.

Previous studies show that prevalence of ERP varies from ~1% to 25% in a general population [10, 13, 14, 16]. This wide range of ERP prevalence reflects differences in age range, proportions of male subjects, and inconsistent definition of ERP between studies. In the MONICA/KORA study that included 6,213 participants between 35-74 years, ERP prevalence of individuals above 55 years was 6.29% [15]. In the Japanese NIPPON DATA90 study with 7,630 participants aged 30-95 years, ERP prevalence of individuals above 60 years was 2.5% [14]. The number of participants in the older middle-aged and elderly population (aged 55-103 years) was highest in our study compared to previous studies. The prevalence of ERP in this age group was 19.2%— the highest overall ERP prevalence among all community-based studies that included study subjects with a mean age > 55 years. This may suggest that aging causes degeneration of the cardiac conduction pathways [21] or increased fibrosis and fat deposition within the heart of elderly patients [22].

In the past years, the association between ERP and mortality has been tested in general populations by several epidemiologic studies [13, 15, 23–25]. The results are inconsistent and conflicting. The MONICA/KORA study screened 6,213 individuals aged 35-74 years and showed increased all-cause and cardiovascular mortality in individuals with ERP [15]. The CARDIA study enrolled 5,039 biracial young adults aged 18-30 years with a follow-up time >20 years and demonstrated that ERP was not associated with all-cause and cardiovascular mortality [13]. The CHD study consisting of 10,864 individuals aged 30-59 years concluded that ERP-positive in the inferior leads was associated with increased risk of cardiac mortality, but was not related to all-cause mortality [25]. A meta-analysis of 16 studies including 4 case-control and 12 prospective or retrospective studies (334,524 individuals from multiple ethnicities) showed that ERP was an electrocardiographic risk marker for deaths related to arrhythmia, cardiac diseases, and all-causes [23, 24]. However, there was considerable heterogeneity because of differences in study designs, ethnicity and potential misdiagnosis of ERP. Moreover, majority of these duties did not analyze older middle-aged and geriatric individuals. In the Japanese NIPPON DATA90 study of 2,433 individuals older than 60 years, subgroup analysis showed that J-point elevation was not associated with cardiovascular death or death from coronary artery disease [14]. Our random-sampling community-based cohort study specifically enrolled older middle-aged and elderly individuals from the Han Chinese population, and prospectively followed up these individuals for 11years with less than 1% dropouts. Our analysis showed that ERP was not associated with both all-cause and cardiovascular mortality.

ECG is globally used as an inexpensive and noninvasive technique to detect electric abnormalities of the heart. Several individuals receive annual health examinations including ECG. ERP is an incidental and common finding on an ECG during a routine health examination. Our study provides an important reference for clinicians or health care providers when they encounter asymptomatic elderly individuals with an incidental ERP and without a family history of SCD in members younger than 40 years.

The results of the association between ERP and mortality in several epidemiologic studies are inconsistent and conflicting [13, 15, 23–25]. The possible reasons may be due to heterogeneity in observational studies, differences in study designs, as well as differences in age and ethnicity of the study subjects. Therefore, prospective long-term randomized clinical trials (RCTs) in different age groups (30-40 year or 60-70 year-olds) in specific ethnic populations are required in the future to minimize all possible confounding factors. Moreover, combining ERP with other ECG risk parameters (such as QTc interval) may provide better risk stratification of large community-based cohorts compared to ERP as a single ECG risk factor.

There are several limitations in our study. Firstly, our findings may not apply to younger individuals because our study consisted of individuals above the age of 55 years. Secondly, the HALST cohort was restricted to individuals of Han Chinese ethnicity, and may not be applicable to other ethnicities. Thirdly, detailed clinical information including echocardiographic assessments, coronary angiography, and medications were not available in the HALST database because this data was obtained during screening.

In summary, our study shows that prevalence of ERP in a standard 12-lead ECG is common in relatively healthy, community-dwelling, ambulatory individuals above 55 years. Moreover, ERP alone is not associated with all-cause and cardiovascular mortality.

## MATERIALS AND METHODS

### Study subjects and inclusion criteria

Majority (>95%) of Taiwanese are of Han Chinese ancestry and nearly 2% are of aboriginal Austronesian ancestry [26]. In this study, we initially included 5,380 Han Chinese individuals of the HALST study cohort and excluded all aboriginal Taiwanese subjects [27–29]. This study was approved by the Ethics Committee of the National Health Research Institutes and conducted according to the principles of the Declaration of Helsinki. We obtained signed informed consent from the study subjects. The study cohort represented a random sample of the entire national population and was selected based on citizen IDs from 7 communities in the Northern, Central, Southern, and Eastern regions of Taiwan. The eligible and willing participants were enrolled from December 2008 to March 2013. At the time of enrollment, we prospectively collected three serial 12-lead ECGs and other relevant clinical and demographic information from the study subjects.

We then excluded individuals with cancer, significant heart diseases such as myocardial infarction and severe valvular diseases, ventricular conduction delay (left or right bundle branch block or QRS>120 ms), atrial fibrillation or flutter, QTc interval corrected for heart rate with Bazett's formula (QTc) of less than 340 msec (short QT interval) or more than 440 msec (long QT interval) at baseline and before arrhythmia [30, 31], Brugada type 1 ECG [32], catecholaminergic arrhythmias [33], ventricular pre-excitation, and implanted pacemakers. The final study cohort consisted of 4,615 individuals, which was followed up for 95.1±21.9 months. During follow-up, 770 study subjects died of cardiac (n=87) or other causes (n=683).

All participants were prospectively followed on a regular basis according to the HALST study framework guidelines and the follow-up information was available until April 2019. We obtained death certificates from the National Taiwan Ministry of Health and Welfare and evaluated the cause using the 10^th^ revision of the International Classification of Diseases (ICD-10). The death from cardiac causes was defined by ICD codes, I01-I02.0, I05-I09, I20-I25, I27, and I30-I52.

### ECG recording and definition of ERP

The 12-lead ECGs (Hewlett Packard, USA) were recorded using standard settings of 10 mm/mV and 25 mm/s. PR and QRS were computed automatically, whereas, QTc was computed using the Bazett’s formula [34]. ERP was defined as a J-point (QRS-ST junction) elevation of at least ≥0.1 mV from baseline in ≥2 adjacent leads with either QRS slurring or notching morphology, as described by Haissaguerre et al. and the 2015 consensus criteria [7, 35]. The anterior precordial leads (V1 to V3) were excluded from the analysis to avoid inclusion of patients with right ventricular arrhythmogenic dysplasia or the Brugada syndrome [36, 37].

ECGs were displayed on a 24-inch computer screen in multiple formats to enable careful classification of slurring on the downslope of the R and J waves. All ECGs were independently analyzed and interpreted in a random order by two trained cardiologists who were blinded to clinical data and the follow-up status. In case of divergent results, a third blinded cardiologist re-interpreted the ECG and a preliminary ERP status was achieved by a majority vote. After the preliminary decision on ERP status, two trained cardiologists jointly reassessed all the ECGs that were considered ERP-positive and reached a final consensus decision on the ERP status.

### Long-term prognosis and follow-up

We collected clinical data regarding history of unexplained syncope, circumstances of sudden cardiac arrest, and a family history of unexplained sudden death (at <40 years of age).

The primary end point was death from cardiac causes, and the secondary end point was death from any cause before April 2019. An annual follow-up telephone interview of all the study participants was carried out by the study nurses to determine any new cardiovascular or frailty-related events after the initial enrollment. The cause of deaths was determined by examining the death certificates from the National Taiwan Institute of Health and Welfare and reviewed by 2 clinicians blinded to the ECG results. The cause of sudden death from arrhythmia was adjudicated by a committee of experienced cardiologists who were blinded to the data from the ECG analyses.

### Statistical analysis

We used the Fisher exact test to compare categorical variables and Student’s t test to compare continuous data between the groups. Linear regression was used for continuous variables and logistic regression was used for dichotomous variables to determine the relationship between ERP and mortality. Multivariate analysis was performed to determine hazard ratios (HRs) and confidence intervals (CIs). The models were primarily adjusted for age and sex, and further adjusted for covariates that were selected on the basis of previous evidence of an association with death from cardiovascular causes or other causes. This included continuous variables such as BMI, SBP, DBP, and QTc, and categorical variables such as smoking status (current, quit, never), hypertension, diabetes mellitus, hyperlipidemia, stroke, and chronic kidney disease. The relationship between ERP and mortality was calculated using a weighted Cox proportional hazards model [38]. Kaplan-Meier survival curves and log rank tests were used to determine survival times of different groups of individuals. Clinical factors such as gender, age, smoking, SBP, DBP, BMI, stroke, diabetes mellitus, hypertension, hyperlipidemia, and chronic kidney disease were used for multivariable analysis. P < 0.05 was considered statistically significant. Inter-observer agreement was based on the overall proportion of agreements and the Kappa statistic across all ECGs. Statistical analyses were performed using the R software, version 4.0.1 (R Foundation for Statistical Computing) [39].

## Supplementary Material

Supplementary Materials

Supplementary Figures
