# Supplementary material for: Role of electrocardiographic early repolarization pattern in long-term outcomes of a community-based middle-aged and geriatric ambulatory population: a prospective cohort study
Source: Aging (Albany NY). 2020 Dec 19;12(24):26140–87. doi: 10.18632/aging.202369 (PMC7803508; doi:10.18632/aging.202369)
Supplement: Supplementary Materials [file aging-12-202369-s001.pdf]

SUPPLEMENTARY MATERIALS

Abbreviations

BMI: body mass index; BP: blood pressure; CI: confidence interval; ERP: early repolarization pattern by the criteria adopted in 2015 [1]; HR: hazard ratio; QTcB: corrected QT interval (QTc) calculated by Bazett's equations.

inf^+ indicates ERP-positive in the inferior leads

lat^+ indicates ERP-positive in the lateral leads

inf^+ lat^+ indicates ERP-positive in both inferior and lateral leads

Supplementary References

1. Macfarlane PW, Antzelevitch C, Haissaguerre M, Huikuri HV, Potse M, Rosso R, Sacher F, Tikkanen JT, Wellens H, Yan GX. The Early Repolarization Pattern: A Consensus Paper. J Am Coll Cardiol. 2015; 66:470–77. https://doi.org/10.1016/j.jacc.2015.05.033 PMID:26205599
