# Supplementary material for: Role of electrocardiographic early repolarization pattern in long-term outcomes of a community-based middle-aged and geriatric ambulatory population: a prospective cohort study
Source: Aging (Albany NY). 2020 Dec 19;12(24):26140–87. doi: 10.18632/aging.202369 (PMC7803508; doi:10.18632/aging.202369)
Supplement: Supplementary Figures [file aging-12-202369-s002.pdf]

SUPPLEMENTARY FIGURES

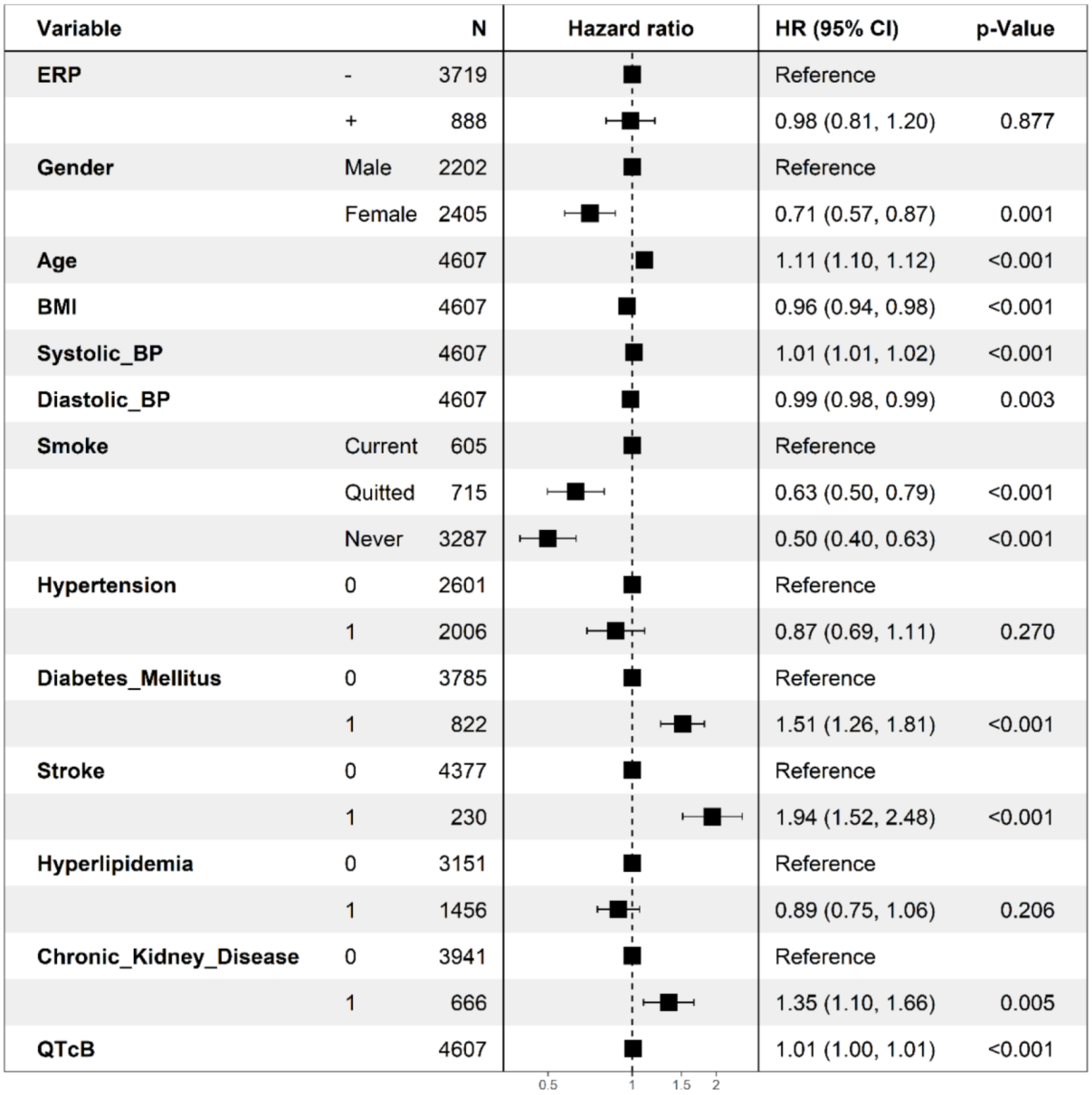

Variable		N	HR (95% CI)	p-Value
ERP	-	3719	Reference	
	+	888	0.98 (0.81, 1.20)	0.877
Gender	Male	2202	Reference	
	Female	2405	0.71 (0.57, 0.87)	0.001
Age		4607	1.11 (1.10, 1.12)	<0.001
BMI		4607	0.96 (0.94, 0.98)	<0.001
Systolic_BP		4607	1.01 (1.01, 1.02)	<0.001
Diastolic_BP		4607	0.99 (0.98, 0.99)	0.003
Smoke	Current	605	Reference	
	Quitted	715	0.63 (0.50, 0.79)	<0.001
	Never	3287	0.50 (0.40, 0.63)	<0.001
Hypertension	0	2601	Reference	
	1	2006	0.87 (0.69, 1.11)	0.270
Diabetes_Mellitus	0	3785	Reference	
	1	822	1.51 (1.26, 1.81)	<0.001
Stroke	0	4377	Reference	
	1	230	1.94 (1.52, 2.48)	<0.001
Hyperlipidemia	0	3151	Reference	
	1	1456	0.89 (0.75, 1.06)	0.206
Chronic_Kidney_Disease	0	3941	Reference	
	1	666	1.35 (1.10, 1.66)	0.005
QTcB		4607	1.01 (1.00, 1.01)	<0.001

Supplementary Figure 1. Association between ERP and all-cause mortality. The hazard ratios and *P*-values were calculated by the Cox proportional hazards model adjusted for all covariables.

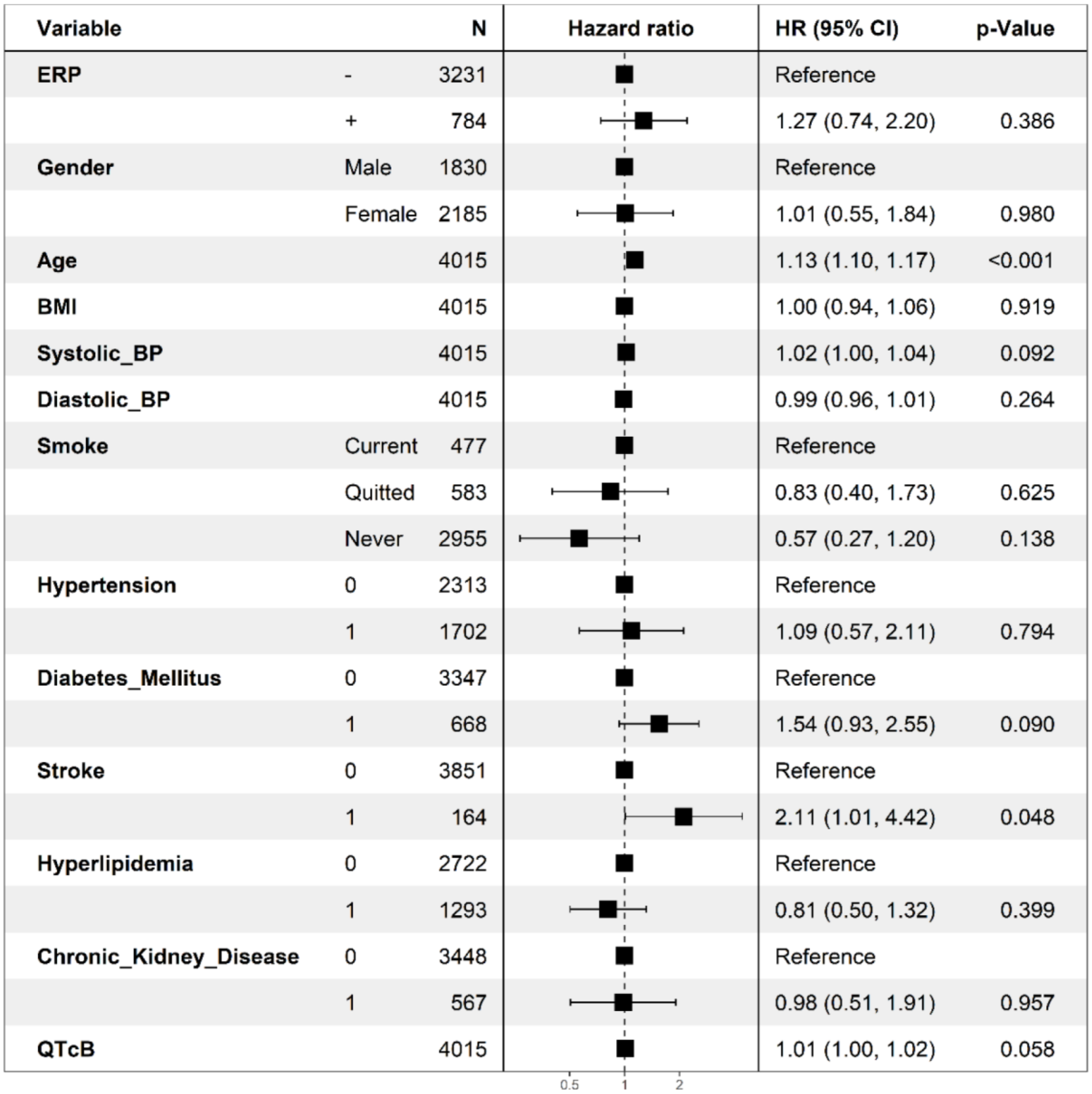

Variable		N	HR (95% CI)	p-Value
**ERP**	-	3231	Reference	
	+	784	1.27 (0.74, 2.20)	0.386
**Gender**	Male	1830	Reference	
	Female	2185	1.01 (0.55, 1.84)	0.980
**Age**		4015	1.13 (1.10, 1.17)	<0.001
**BMI**		4015	1.00 (0.94, 1.06)	0.919
**Systolic_BP**		4015	1.02 (1.00, 1.04)	0.092
**Diastolic_BP**		4015	0.99 (0.96, 1.01)	0.264
**Smoke**	Current	477	Reference	
	Quitted	583	0.83 (0.40, 1.73)	0.625
	Never	2955	0.57 (0.27, 1.20)	0.138
**Hypertension**	0	2313	Reference	
	1	1702	1.09 (0.57, 2.11)	0.794
**Diabetes_Mellitus**	0	3347	Reference	
	1	668	1.54 (0.93, 2.55)	0.090
**Stroke**	0	3851	Reference	
	1	164	2.11 (1.01, 4.42)	0.048
**Hyperlipidemia**	0	2722	Reference	
	1	1293	0.81 (0.50, 1.32)	0.399
**Chronic_Kidney_Disease**	0	3448	Reference	
	1	567	0.98 (0.51, 1.91)	0.957
**QTcB**		4015	1.01 (1.00, 1.02)	0.058

Supplementary Figure 2. Association between ERP and cardiovascular mortality. The hazard ratios and *P*-values were calculated by the Cox proportional hazards model adjusted for all covariables.

Variable		N	HR (95% CI)	p-Value
**ERP**	-	3719	Reference	
	inf. +	573	0.93 (0.74, 1.17)	0.534
**Gender**	Male	2038	Reference	
	Female	2254	0.72 (0.58, 0.89)	0.002
**Age**		4292	1.11 (1.10, 1.12)	<0.001
**BMI**		4292	0.95 (0.93, 0.98)	<0.001
**Systolic_BP**		4292	1.01 (1.01, 1.02)	<0.001
**Diastolic_BP**		4292	0.99 (0.98, 1.00)	0.004
**Smoke**	Current	554	Reference	
	Quitted	674	0.63 (0.49, 0.80)	<0.001
	Never	3064	0.49 (0.39, 0.63)	<0.001
**Hypertension**	0	2407	Reference	
	1	1885	0.88 (0.69, 1.12)	0.295
**Diabetes_Mellitus**	0	3517	Reference	
	1	775	1.54 (1.28, 1.85)	<0.001
**Stroke**	0	4072	Reference	
	1	220	1.97 (1.54, 2.53)	<0.001
**Hyperlipidemia**	0	2927	Reference	
	1	1365	0.88 (0.74, 1.06)	0.182
**Chronic_Kidney_Disease**	0	3670	Reference	
	1	622	1.36 (1.10, 1.69)	0.004
**QTcB**		4292	1.01 (1.00, 1.01)	<0.001

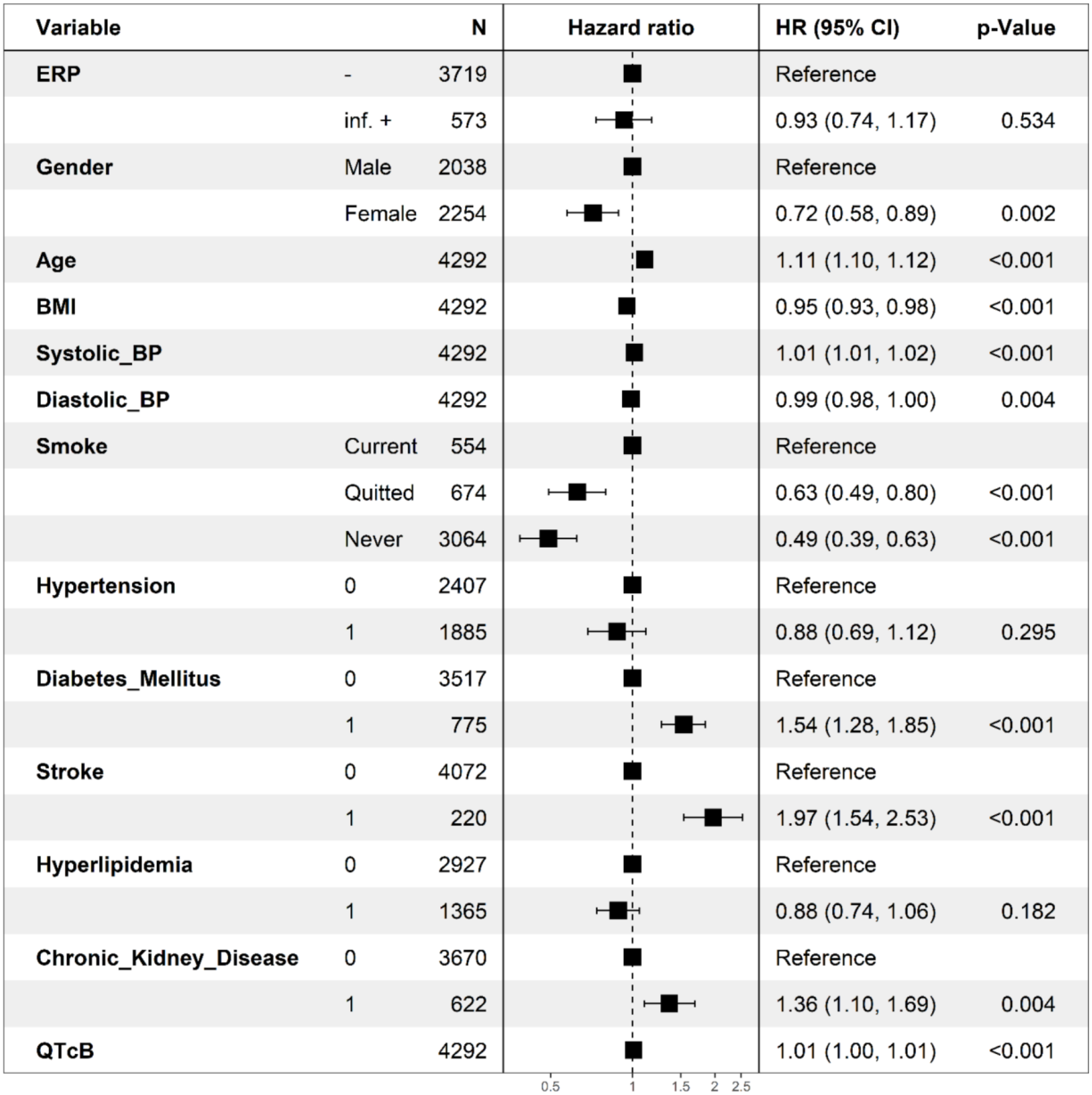

Supplementary Figure 3. Association between positive ERP in the inferior leads and all-cause mortality. The hazard ratios and *P*-values were calculated by the Cox proportional hazards model adjusted for all covariables.

Variable		N	Hazard ratio	HR (95% CI)	p-Value
**ERP**	-	3231	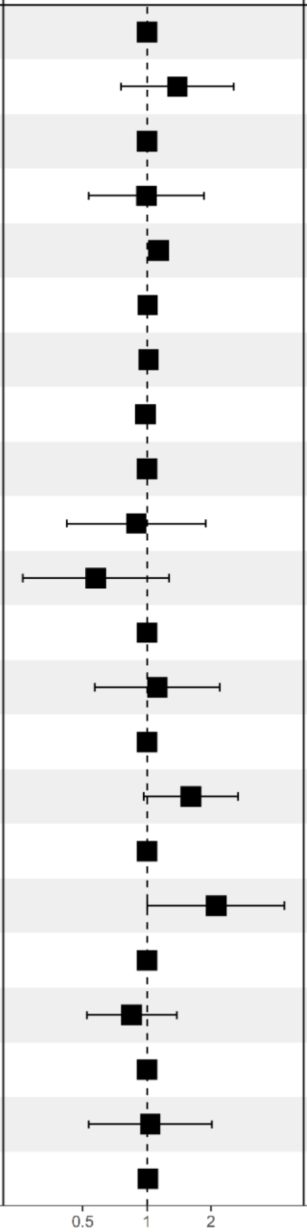	Reference	
	inf. +	502		1.39 (0.76, 2.55)	0.288
**Gender**	Male	1687		Reference	
	Female	2046		0.99 (0.53, 1.86)	0.987
**Age**		3733		1.13 (1.10, 1.17)	<0.001
**BMI**		3733		1.01 (0.94, 1.07)	0.843
**Systolic_BP**		3733		1.02 (1.00, 1.04)	0.051
**Diastolic_BP**		3733		0.99 (0.96, 1.01)	0.271
**Smoke**	Current	433		Reference	
	Quitted	548		0.89 (0.42, 1.89)	0.764
	Never	2752		0.58 (0.26, 1.27)	0.170
**Hypertension**	0	2137		Reference	
	1	1596		1.12 (0.57, 2.19)	0.743
**Diabetes_Mellitus**	0	3104		Reference	
	1	629		1.61 (0.97, 2.67)	0.067
**Stroke**	0	3577		Reference	
	1	156		2.11 (1.01, 4.43)	0.048
**Hyperlipidemia**	0	2521		Reference	
	1	1212		0.85 (0.52, 1.38)	0.504
**Chronic_Kidney_Disease**	0	3204		Reference	
	1	529		1.04 (0.53, 2.02)	0.918
**QTcB**		3733		1.01 (1.00, 1.02)	0.026

Supplementary Figure 4. Association between positive ERP in the inferior leads and cardiovascular mortality. The hazard ratios and *P*-values were calculated by the Cox proportional hazards model after adjusting for covariables.

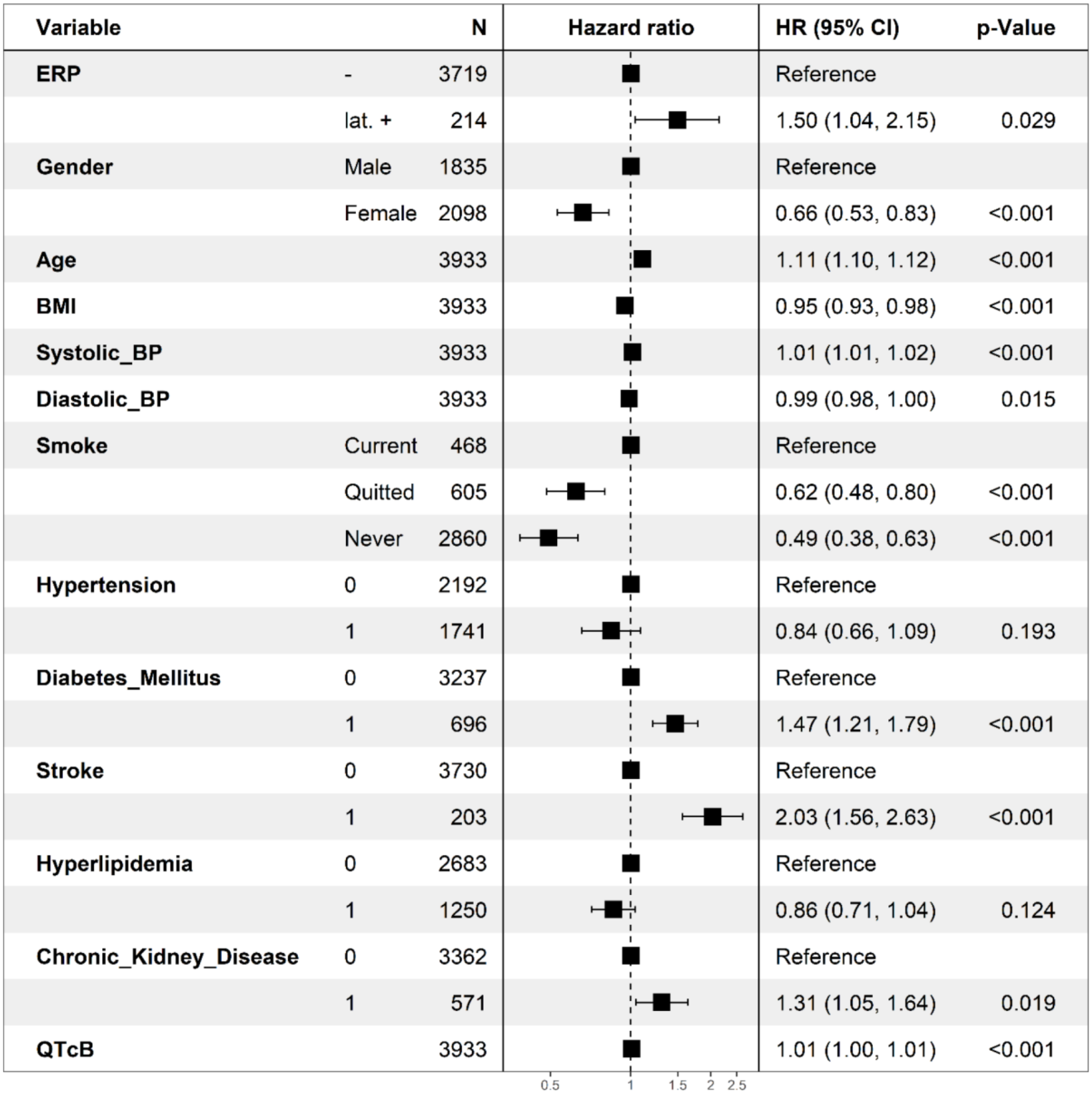

Supplementary Figure 5. Association between ERP-positive in the lateral leads and all-cause mortality. The hazard ratios and *P*-values were calculated by the Cox proportional hazards model after adjusting for covariables.

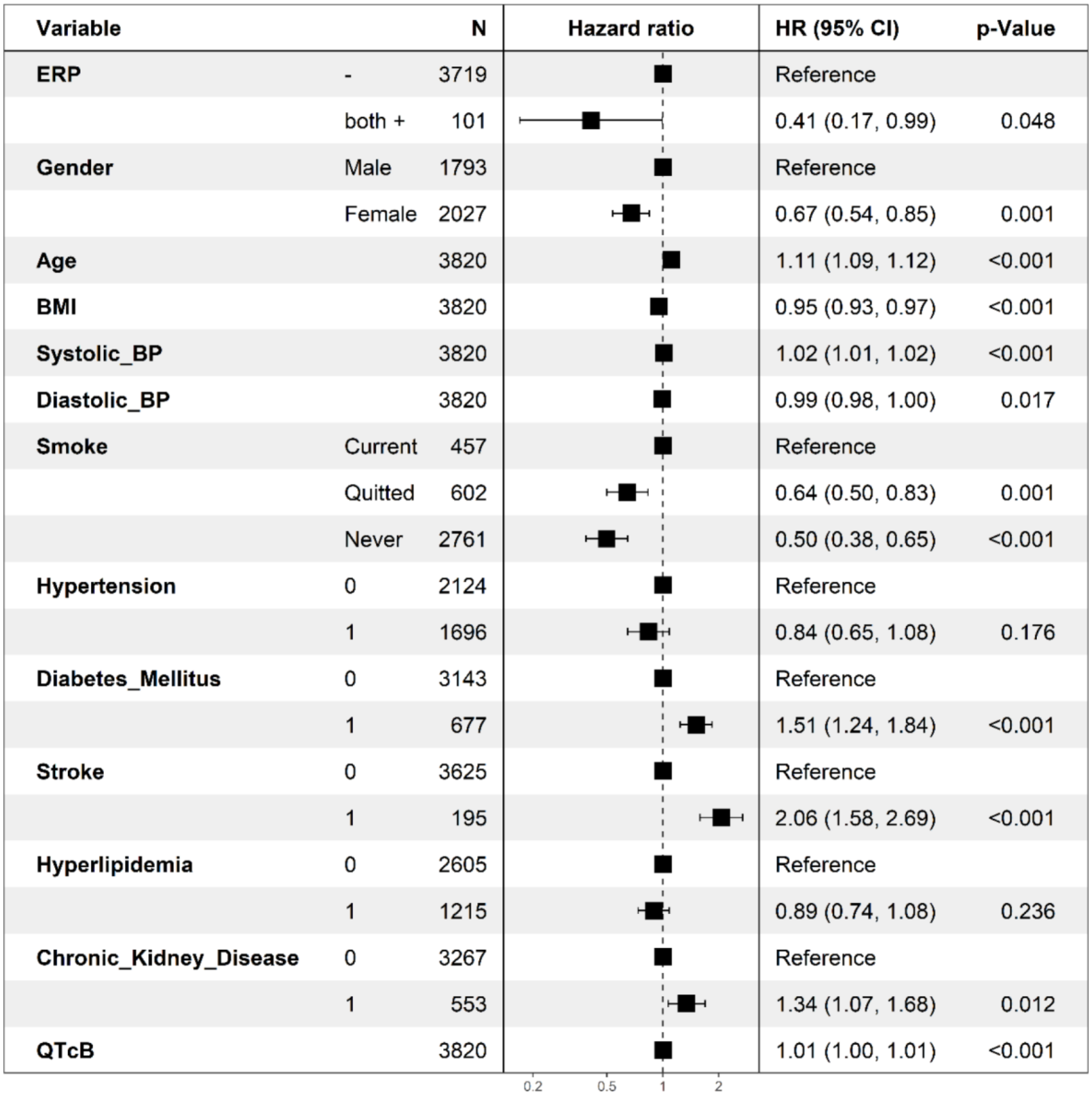

Variable		N	Hazard ratio	HR (95% CI)	p-Value
ERP	-	3719		Reference	
	both +	101		0.41 (0.17, 0.99)	0.048
Gender	Male	1793		Reference	
	Female	2027		0.67 (0.54, 0.85)	0.001
Age		3820		1.11 (1.09, 1.12)	<0.001
BMI		3820		0.95 (0.93, 0.97)	<0.001
Systolic_BP		3820		1.02 (1.01, 1.02)	<0.001
Diastolic_BP		3820		0.99 (0.98, 1.00)	0.017
Smoke	Current	457		Reference	
	Quitted	602		0.64 (0.50, 0.83)	0.001
	Never	2761		0.50 (0.38, 0.65)	<0.001
Hypertension	0	2124		Reference	
	1	1696		0.84 (0.65, 1.08)	0.176
Diabetes_Mellitus	0	3143		Reference	
	1	677		1.51 (1.24, 1.84)	<0.001
Stroke	0	3625		Reference	
	1	195		2.06 (1.58, 2.69)	<0.001
Hyperlipidemia	0	2605		Reference	
	1	1215		0.89 (0.74, 1.08)	0.236
Chronic_Kidney_Disease	0	3267		Reference	
	1	553		1.34 (1.07, 1.68)	0.012
QTcB		3820		1.01 (1.00, 1.01)	<0.001

Supplementary Figure 6. Association between positive ERP in both the inferior and lateral leads and all-cause mortality. The hazard ratios and *P*-values were calculated by the Cox proportional hazards model after adjusting for covariables.

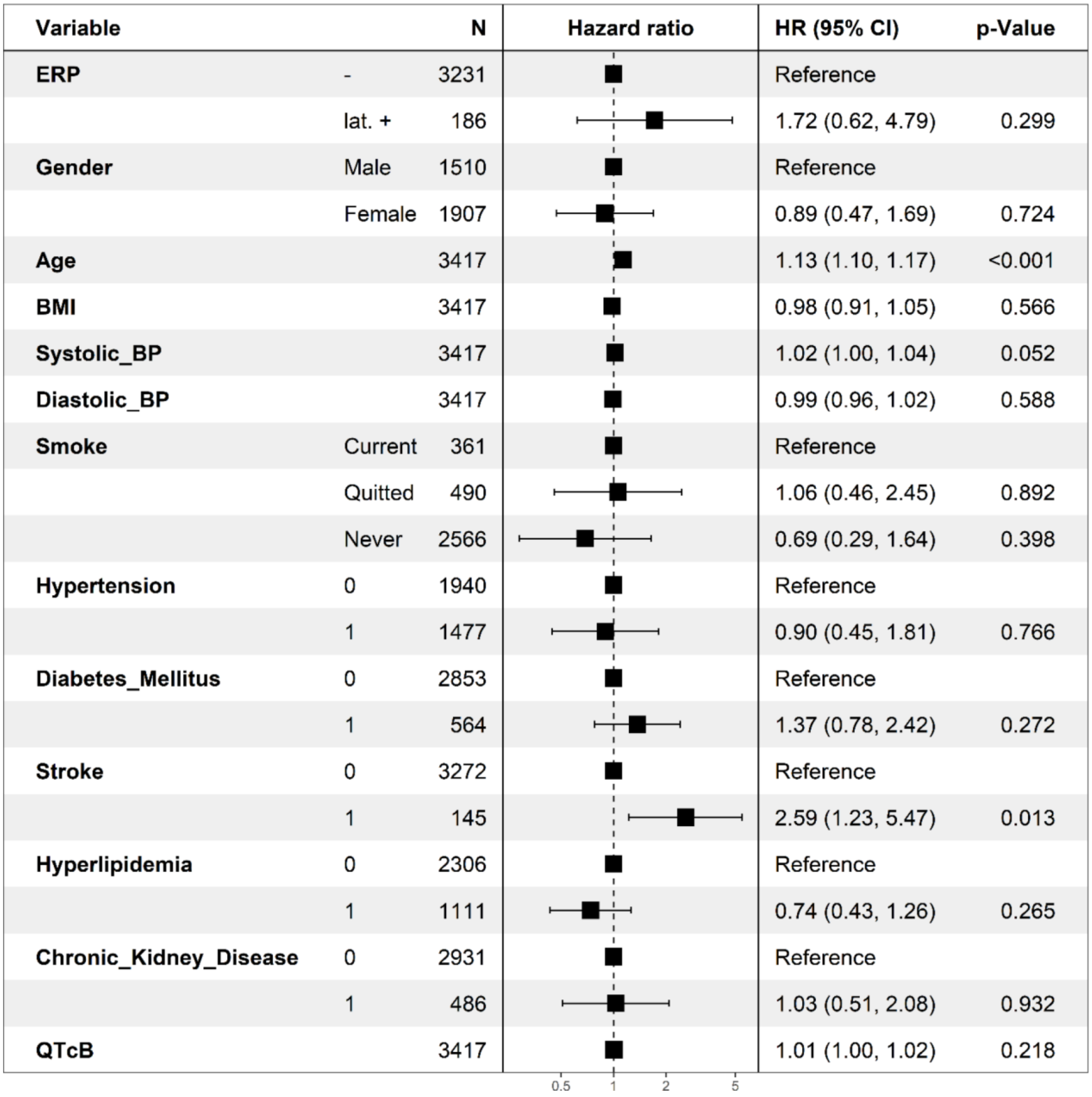

Supplementary Figure 7. Association between positive ERP in the lateral leads and cardiovascular mortality. The hazard ratios and *P*-values were calculated by the Cox proportional hazards model after adjusting for covariables.

Variable		N	HR (95% CI)	p-Value
**ERP**	-	3231	Reference	
	both +	96	0.00 (0.00, Inf)	0.995
**Gender**	Male	1483	Reference	
	Female	1844	0.87 (0.45, 1.68)	0.668
**Age**		3327	1.13 (1.09, 1.17)	<0.001
**BMI**		3327	0.99 (0.93, 1.06)	0.843
**Systolic_BP**		3327	1.02 (1.00, 1.04)	0.019
**Diastolic_BP**		3327	0.99 (0.96, 1.02)	0.607
**Smoke**	Current	355	Reference	
	Quitted	489	1.19 (0.49, 2.86)	0.701
	Never	2483	0.74 (0.29, 1.86)	0.521
**Hypertension**	0	1886	Reference	
	1	1441	0.90 (0.44, 1.84)	0.768
**Diabetes_Mellitus**	0	2778	Reference	
	1	549	1.45 (0.82, 2.56)	0.206
**Stroke**	0	3188	Reference	
	1	139	2.66 (1.26, 5.63)	0.011
**Hyperlipidemia**	0	2249	Reference	
	1	1078	0.78 (0.46, 1.34)	0.365
**Chronic_Kidney_Disease**	0	2855	Reference	
	1	472	1.11 (0.55, 2.25)	0.770
**QTcB**		3327	1.01 (1.00, 1.02)	0.114

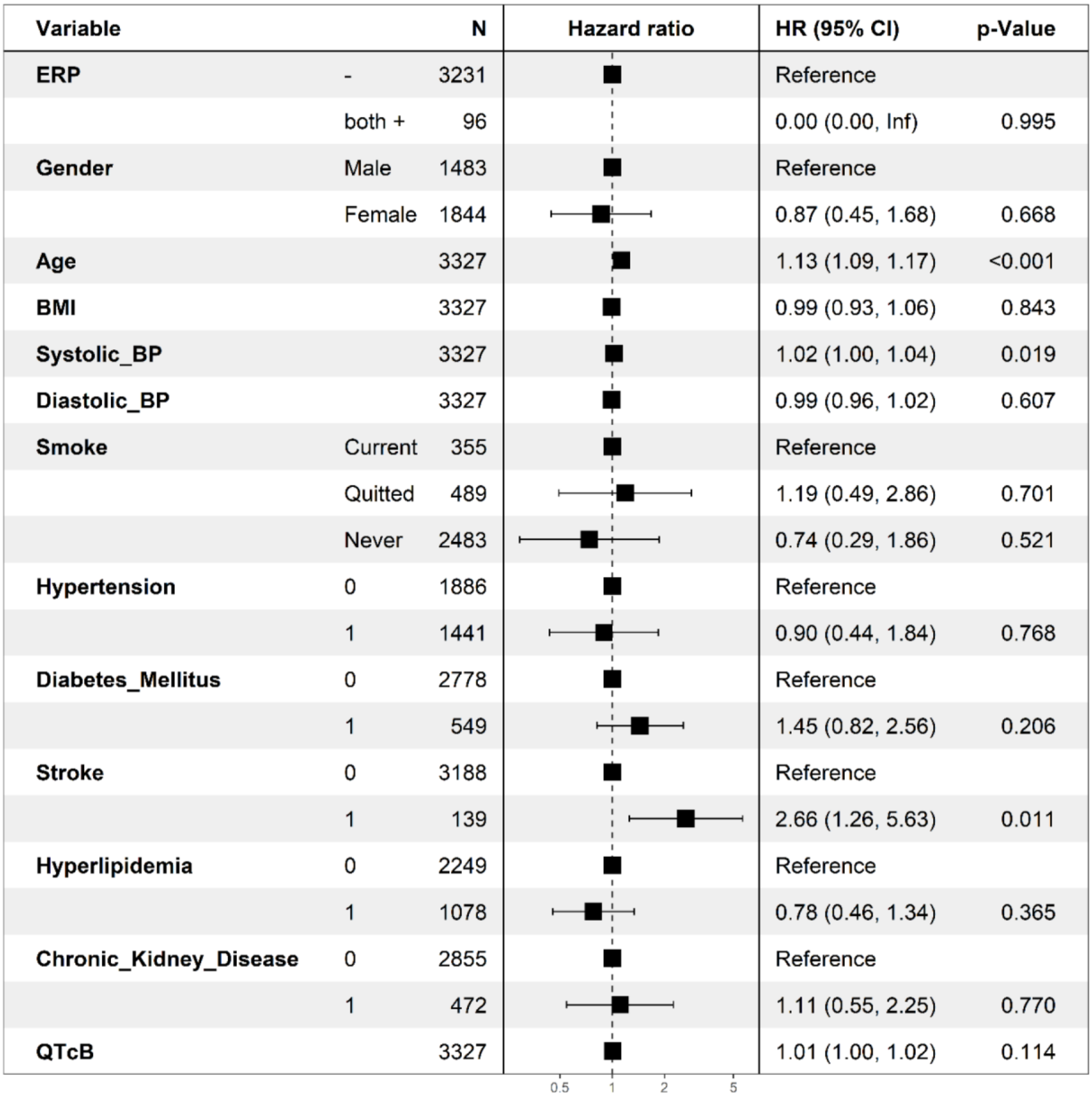

Supplementary Figure 8. Association between positive ERP in both inferior and lateral leads and cardiovascular mortality. The hazard ratios and *P*-values were calculated by the Cox proportional hazards model after adjusting for covariables.

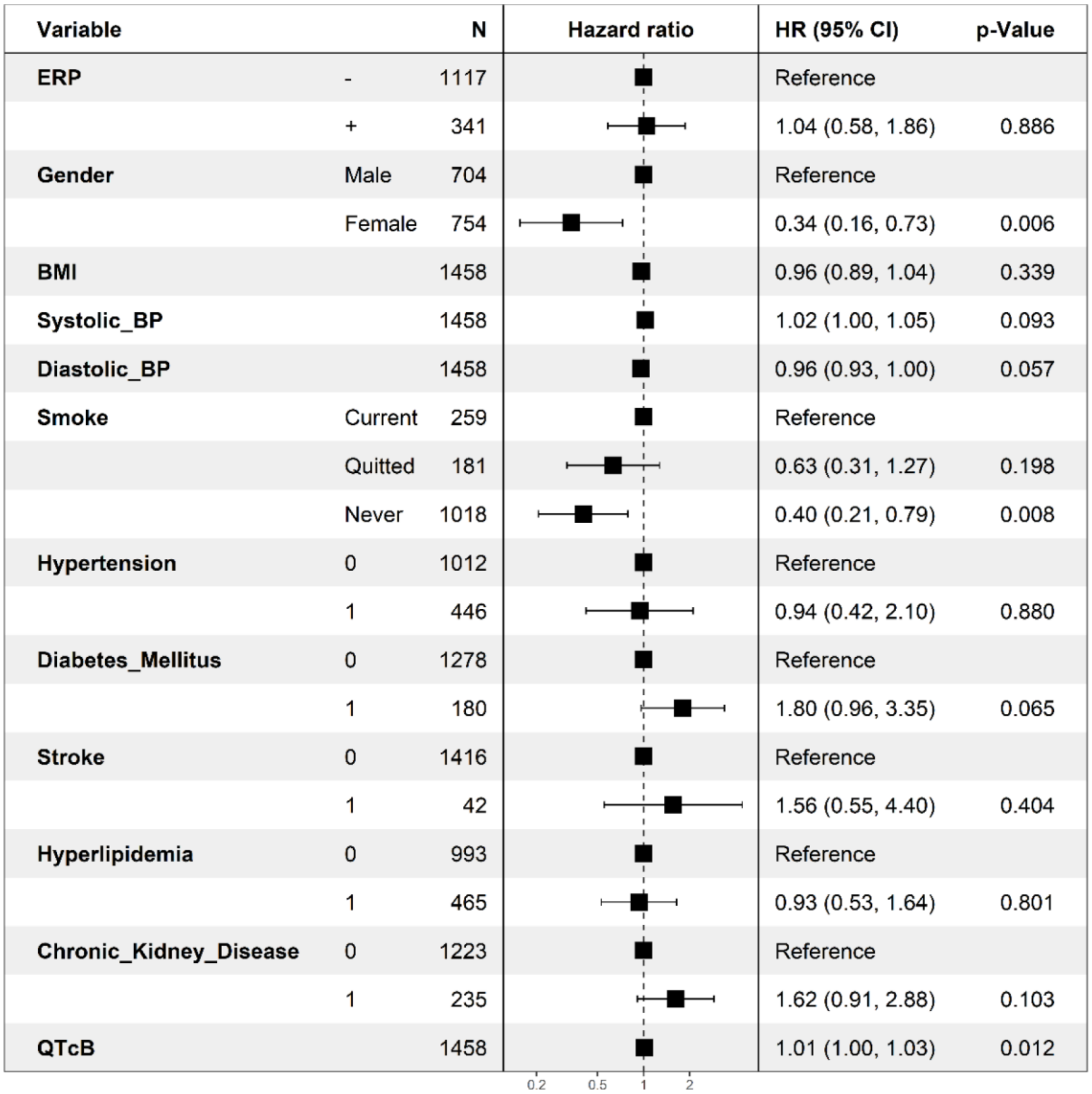

Variable		N	HR (95% CI)	p-Value
**ERP**	-	1117	Reference	
	+	341	1.04 (0.58, 1.86)	0.886
**Gender**	Male	704	Reference	
	Female	754	0.34 (0.16, 0.73)	0.006
**BMI**		1458	0.96 (0.89, 1.04)	0.339
**Systolic_BP**		1458	1.02 (1.00, 1.05)	0.093
**Diastolic_BP**		1458	0.96 (0.93, 1.00)	0.057
**Smoke**	Current	259	Reference	
	Quitted	181	0.63 (0.31, 1.27)	0.198
	Never	1018	0.40 (0.21, 0.79)	0.008
**Hypertension**	0	1012	Reference	
	1	446	0.94 (0.42, 2.10)	0.880
**Diabetes_Mellitus**	0	1278	Reference	
	1	180	1.80 (0.96, 3.35)	0.065
**Stroke**	0	1416	Reference	
	1	42	1.56 (0.55, 4.40)	0.404
**Hyperlipidemia**	0	993	Reference	
	1	465	0.93 (0.53, 1.64)	0.801
**Chronic_Kidney_Disease**	0	1223	Reference	
	1	235	1.62 (0.91, 2.88)	0.103
**QTcB**		1458	1.01 (1.00, 1.03)	0.012

Supplementary Figure 9. Association between ERP and all-cause mortality among individuals aged 55-64 years. The hazard ratios and *P*-values were calculated by the Cox proportional hazards model after adjusting for covariables.

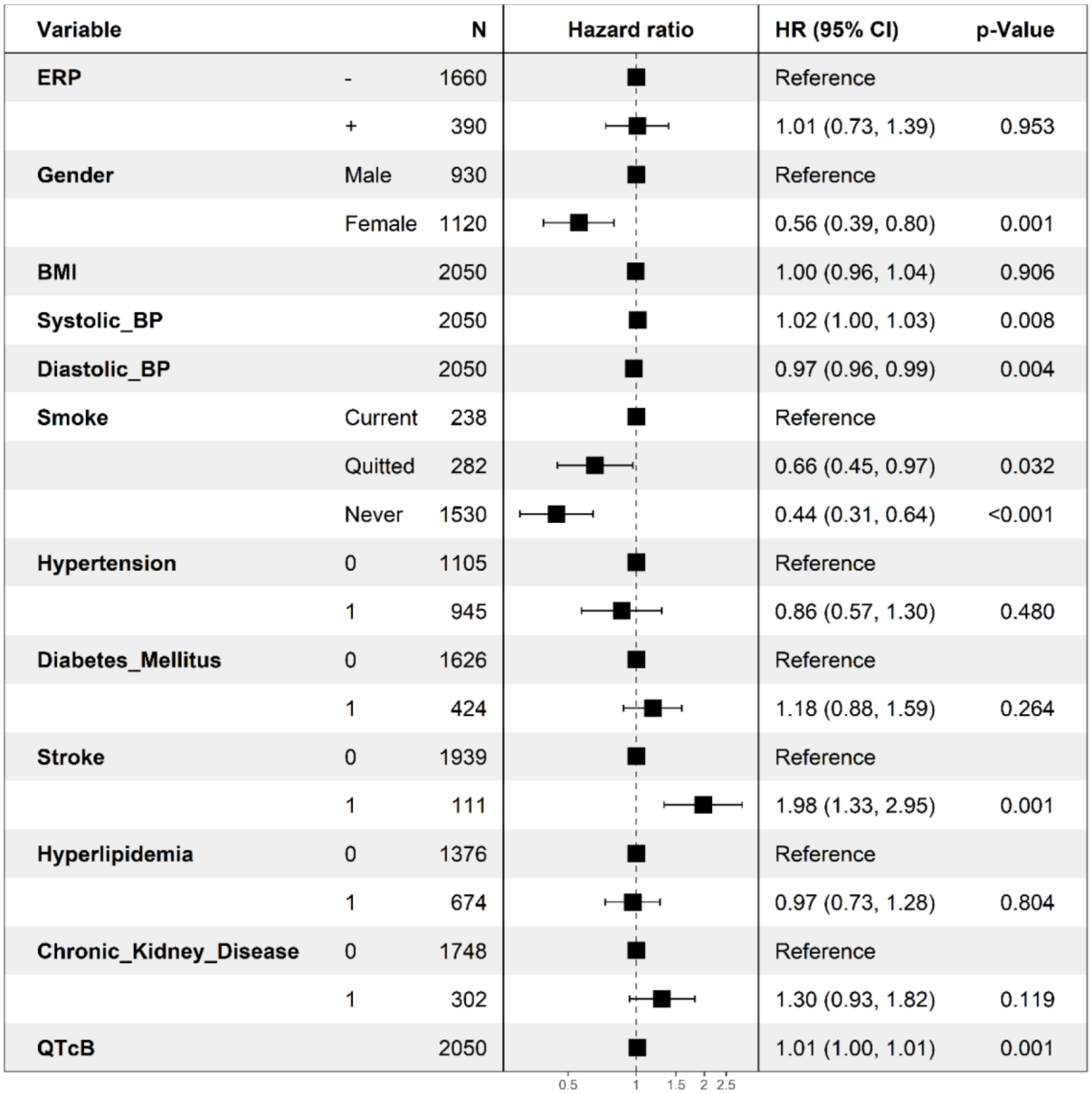

Supplementary Figure 10. Association between ERP and all-cause mortality among individuals aged 65-74 years. The hazard ratios and *P*-values were calculated by the Cox proportional hazards model after adjusting for covariables.

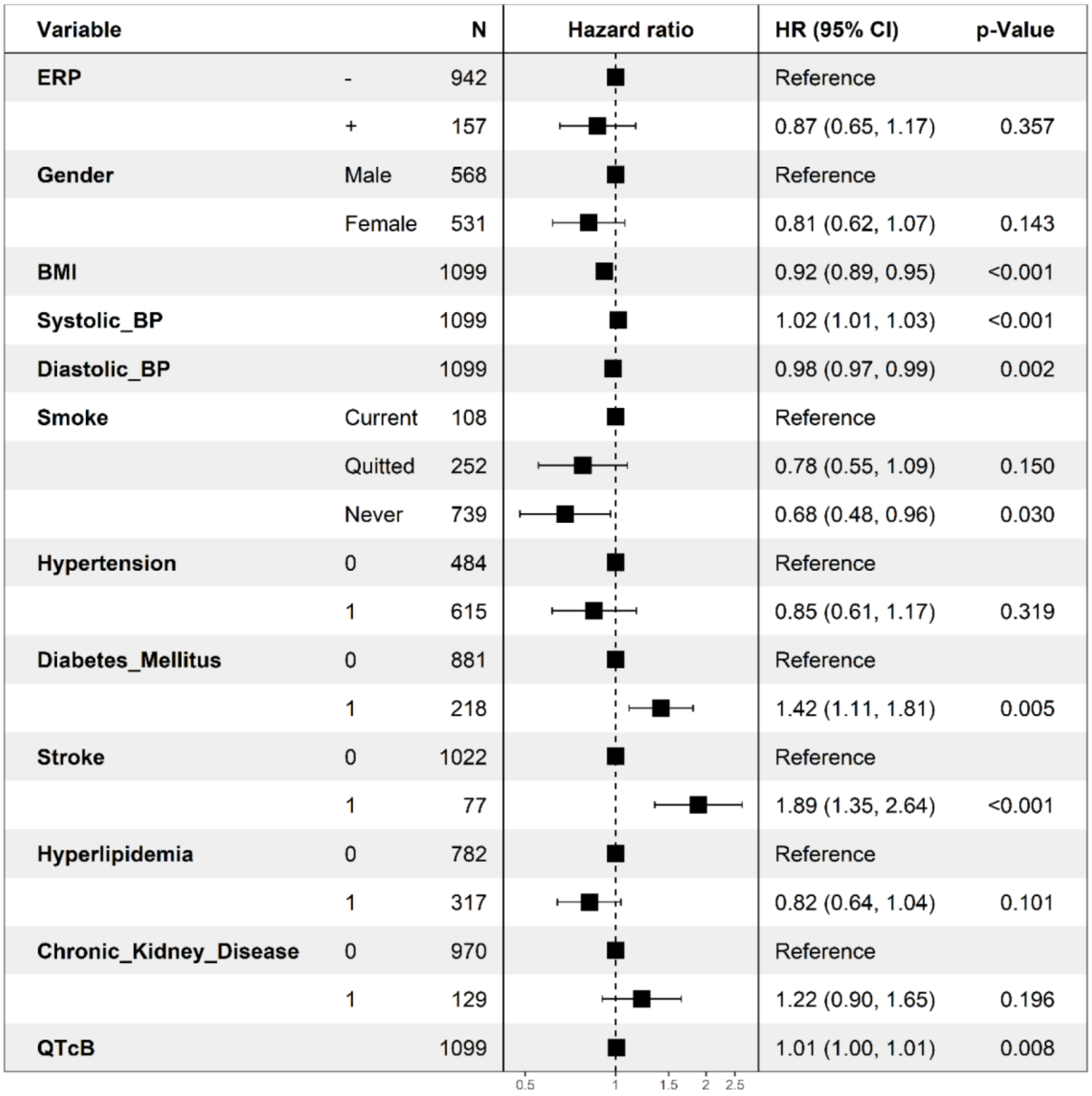

Variable		N	HR (95% CI)	p-Value
**ERP**	-	942	Reference	
	+	157	0.87 (0.65, 1.17)	0.357
**Gender**	Male	568	Reference	
	Female	531	0.81 (0.62, 1.07)	0.143
**BMI**		1099	0.92 (0.89, 0.95)	<0.001
**Systolic_BP**		1099	1.02 (1.01, 1.03)	<0.001
**Diastolic_BP**		1099	0.98 (0.97, 0.99)	0.002
**Smoke**	Current	108	Reference	
	Quitted	252	0.78 (0.55, 1.09)	0.150
	Never	739	0.68 (0.48, 0.96)	0.030
**Hypertension**	0	484	Reference	
	1	615	0.85 (0.61, 1.17)	0.319
**Diabetes_Mellitus**	0	881	Reference	
	1	218	1.42 (1.11, 1.81)	0.005
**Stroke**	0	1022	Reference	
	1	77	1.89 (1.35, 2.64)	<0.001
**Hyperlipidemia**	0	782	Reference	
	1	317	0.82 (0.64, 1.04)	0.101
**Chronic_Kidney_Disease**	0	970	Reference	
	1	129	1.22 (0.90, 1.65)	0.196
**QTcB**		1099	1.01 (1.00, 1.01)	0.008

Supplementary Figure 11. Association between ERP and all-cause mortality among individuals aged ≥75 years. The hazard ratios and *P*-values were calculated by the Cox proportional hazards model after adjusting for covariables.

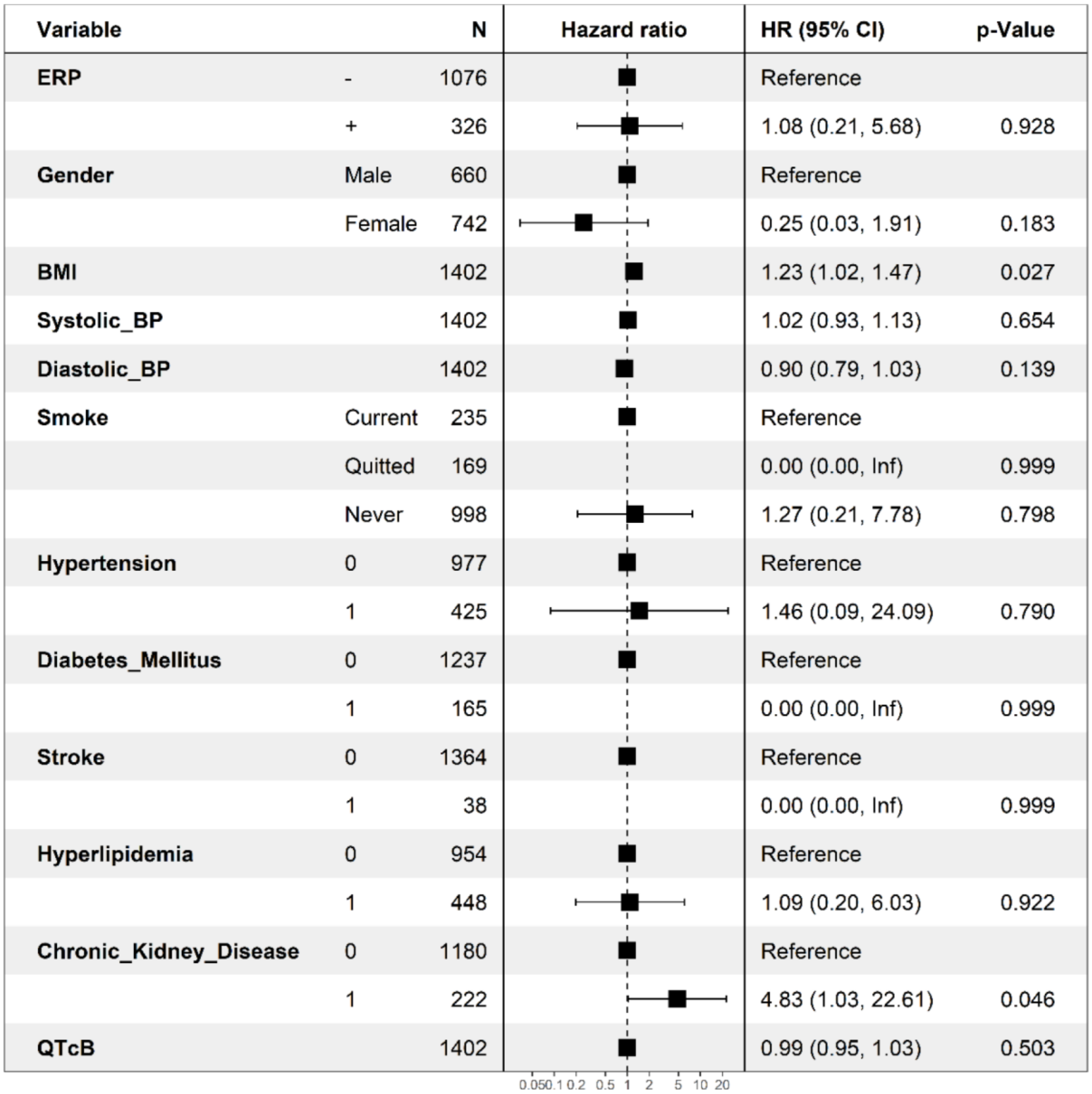

Supplementary Figure 12. Association between ERP and cardiovascular mortality among individuals aged 55-64 years. The hazard ratios and *P*-values were calculated by the Cox proportional hazards model after adjusting for covariables.

Variable		N	HR (95% CI)	p-Value
**ERP**	-	1489	Reference	
	+	346	0.83 (0.28, 2.47)	0.744
**Gender**	Male	792	Reference	
	Female	1043	0.38 (0.13, 1.10)	0.075
**BMI**		1835	1.02 (0.90, 1.15)	0.773
**Systolic_BP**		1835	1.00 (0.96, 1.04)	0.946
**Diastolic_BP**		1835	1.00 (0.95, 1.05)	0.986
**Smoke**	Current	183	Reference	
	Quitted	239	0.89 (0.26, 3.01)	0.850
	Never	1413	0.68 (0.21, 2.19)	0.517
**Hypertension**	0	988	Reference	
	1	847	1.72 (0.50, 5.93)	0.391
**Diabetes_Mellitus**	0	1471	Reference	
	1	364	1.17 (0.47, 2.92)	0.731
**Stroke**	0	1748	Reference	
	1	87	3.59 (1.30, 9.92)	0.014
**Hyperlipidemia**	0	1229	Reference	
	1	606	0.87 (0.37, 2.04)	0.744
**Chronic_Kidney_Disease**	0	1575	Reference	
	1	260	0.26 (0.03, 1.90)	0.182
**QTcB**		1835	1.02 (1.00, 1.04)	0.017

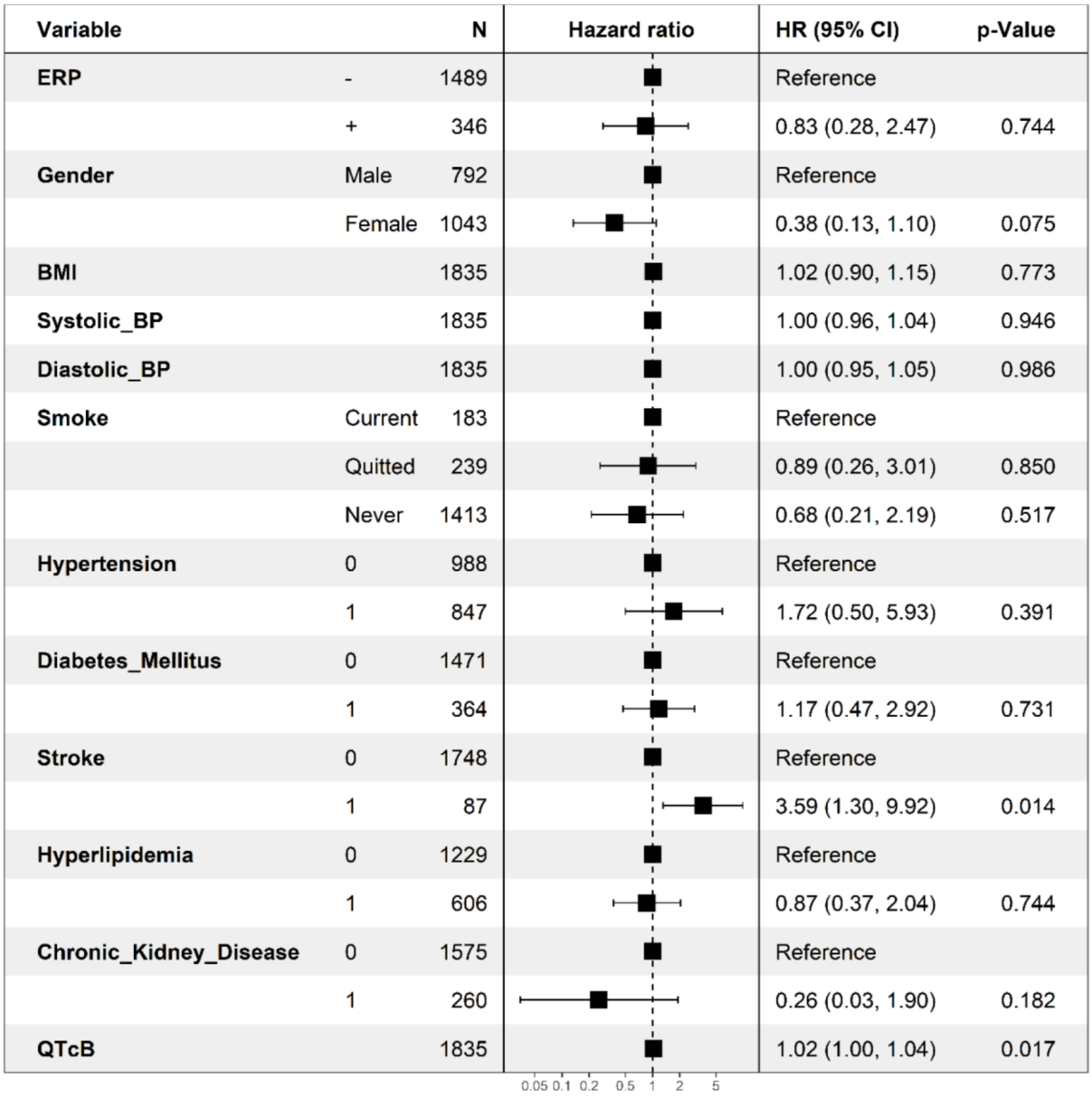

Supplementary Figure 13. Association between ERP and cardiovascular mortality among individuals aged 65-74 years. The hazard ratios and *P*-values were calculated by the Cox proportional hazards model after adjusting for covariables.

Variable		N	HR (95% CI)	p-Value
**ERP**	-	666	Reference	
	+	112	1.54 (0.77, 3.07)	0.219
**Gender**	Male	378	Reference	
	Female	400	1.99 (0.82, 4.82)	0.125
**BMI**		778	0.95 (0.87, 1.03)	0.187
**Systolic_BP**		778	1.03 (1.01, 1.05)	0.014
**Diastolic_BP**		778	0.98 (0.95, 1.01)	0.136
**Smoke**	Current	59	Reference	
	Quitted	175	1.25 (0.44, 3.54)	0.673
	Never	544	0.55 (0.17, 1.78)	0.317
**Hypertension**	0	348	Reference	
	1	430	0.85 (0.36, 2.01)	0.712
**Diabetes_Mellitus**	0	639	Reference	
	1	139	1.77 (0.95, 3.28)	0.070
**Stroke**	0	739	Reference	
	1	39	1.29 (0.40, 4.21)	0.671
**Hyperlipidemia**	0	539	Reference	
	1	239	0.76 (0.41, 1.42)	0.395
**Chronic_Kidney_Disease**	0	693	Reference	
	1	85	1.03 (0.43, 2.44)	0.956
**QTcB**		778	1.01 (1.00, 1.02)	0.193

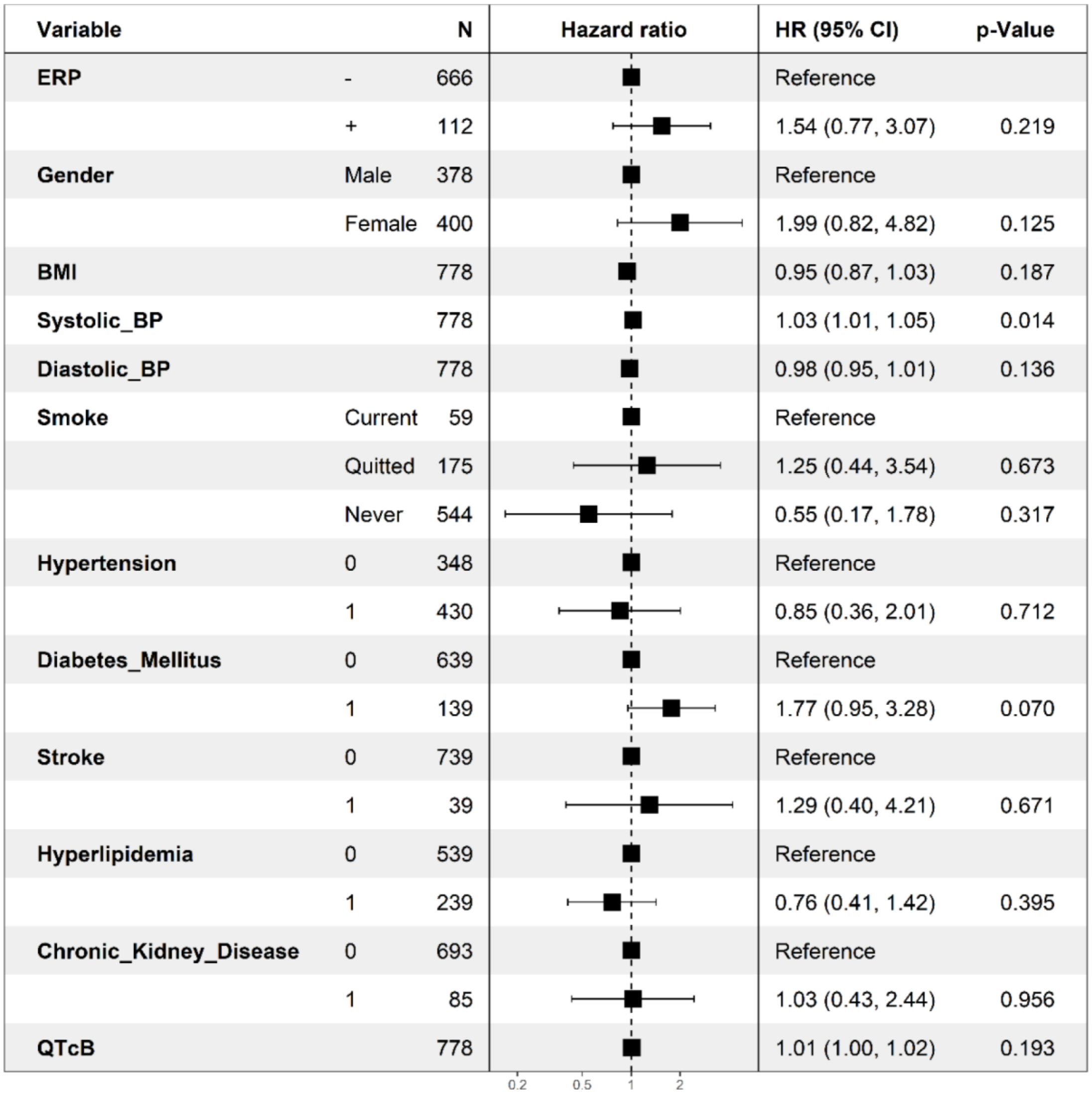

Supplementary Figure 14. Association between ERP and cardiovascular mortality among individuals aged ≥75 years. The hazard ratios and *P*-values were calculated by the Cox proportional hazards model after adjusting for covariables.

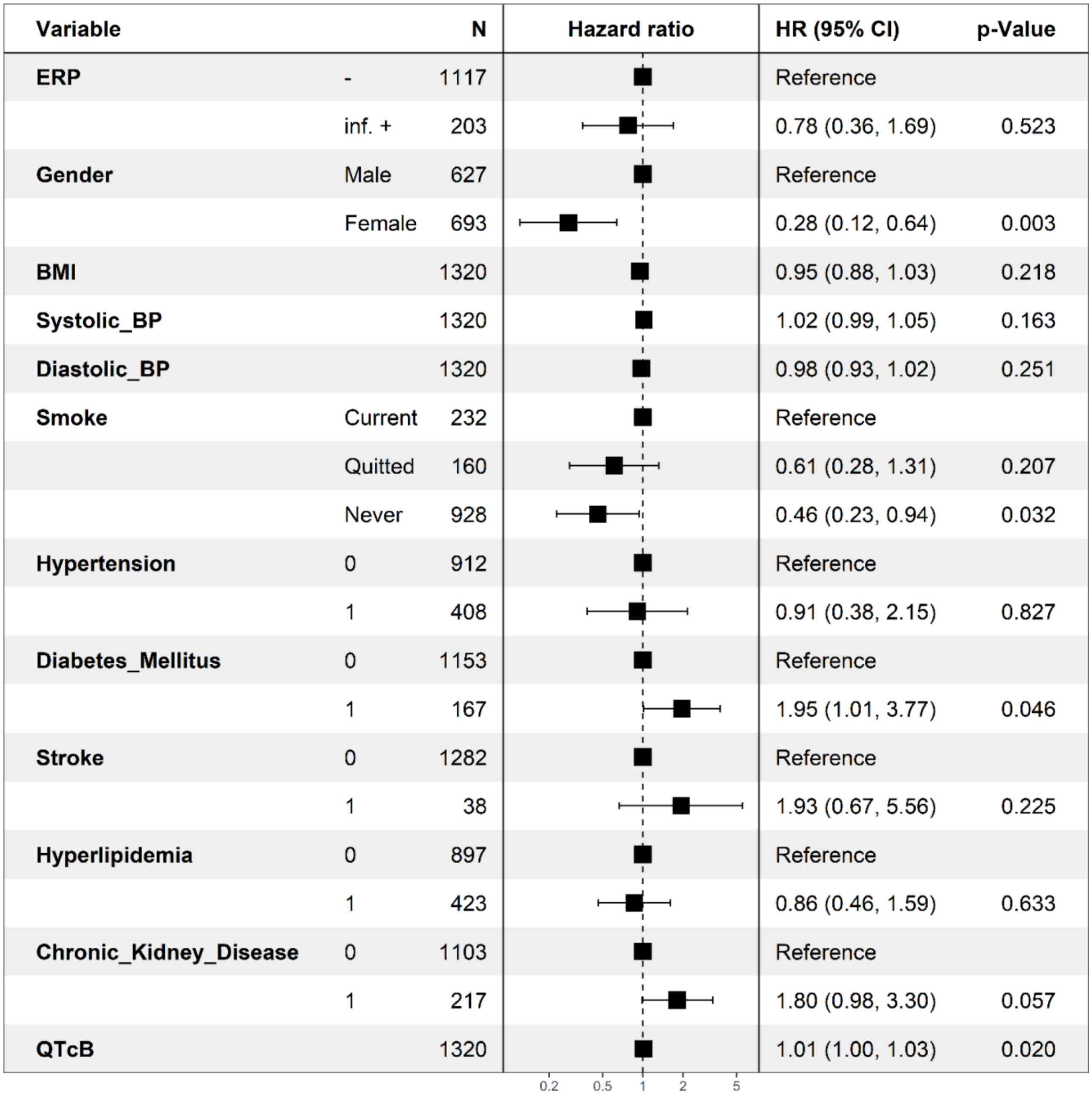

Variable		N	HR (95% CI)	p-Value
**ERP**	-	1117	Reference	
	inf. +	203	0.78 (0.36, 1.69)	0.523
**Gender**	Male	627	Reference	
	Female	693	0.28 (0.12, 0.64)	0.003
**BMI**		1320	0.95 (0.88, 1.03)	0.218
**Systolic_BP**		1320	1.02 (0.99, 1.05)	0.163
**Diastolic_BP**		1320	0.98 (0.93, 1.02)	0.251
**Smoke**	Current	232	Reference	
	Quitted	160	0.61 (0.28, 1.31)	0.207
	Never	928	0.46 (0.23, 0.94)	0.032
**Hypertension**	0	912	Reference	
	1	408	0.91 (0.38, 2.15)	0.827
**Diabetes_Mellitus**	0	1153	Reference	
	1	167	1.95 (1.01, 3.77)	0.046
**Stroke**	0	1282	Reference	
	1	38	1.93 (0.67, 5.56)	0.225
**Hyperlipidemia**	0	897	Reference	
	1	423	0.86 (0.46, 1.59)	0.633
**Chronic_Kidney_Disease**	0	1103	Reference	
	1	217	1.80 (0.98, 3.30)	0.057
**QTcB**		1320	1.01 (1.00, 1.03)	0.020

Supplementary Figure 15. Association between positive ERP in inferior leads and all-cause mortality among individuals aged 55-64 years. The hazard ratios and *P*-values were calculated by the Cox proportional hazards model after adjusting for covariables.

Variable		N	Hazard ratio	HR (95% CI)	p-Value
**ERP**	-	1660		Reference	
	inf. +	249		0.99 (0.68, 1.46)	0.966
**Gender**	Male	859		Reference	
	Female	1050		0.57 (0.39, 0.83)	0.003
**BMI**		1909		0.99 (0.95, 1.03)	0.583
**Systolic_BP**		1909		1.02 (1.00, 1.03)	0.016
**Diastolic_BP**		1909		0.97 (0.95, 0.99)	0.003
**Smoke**	Current	218		Reference	
	Quitted	269		0.64 (0.43, 0.95)	0.026
	Never	1422		0.43 (0.29, 0.64)	<0.001
**Hypertension**	0	1028		Reference	
	1	881		0.91 (0.59, 1.38)	0.646
**Diabetes_Mellitus**	0	1512		Reference	
	1	397		1.17 (0.86, 1.59)	0.329
**Stroke**	0	1803		Reference	
	1	106		2.04 (1.36, 3.06)	0.001
**Hyperlipidemia**	0	1273		Reference	
	1	636		0.97 (0.73, 1.30)	0.859
**Chronic_Kidney_Disease**	0	1627		Reference	
	1	282		1.28 (0.91, 1.81)	0.161
**QTcB**		1909		1.01 (1.00, 1.01)	0.003

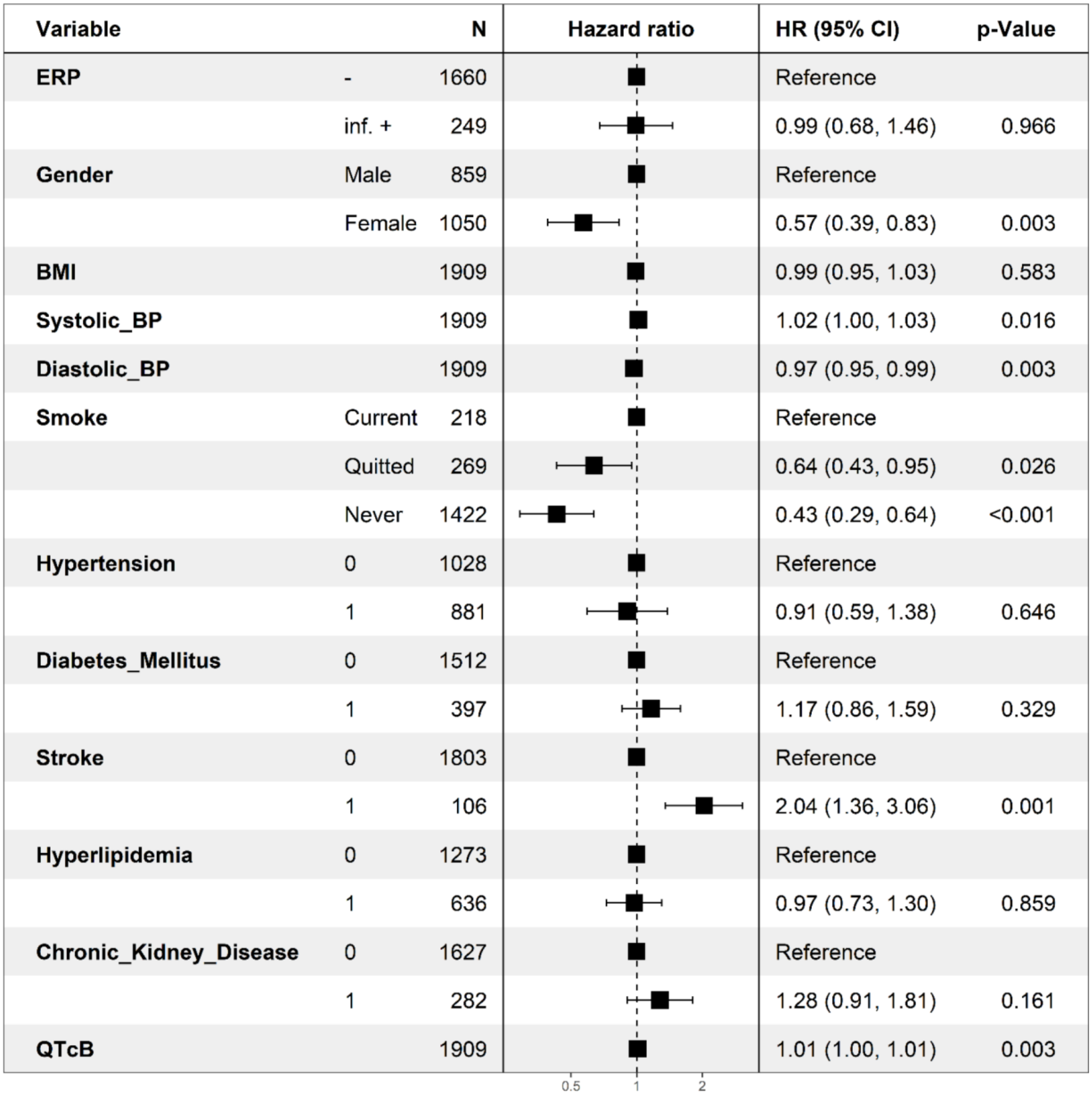

Supplementary Figure 16. Association between positive ERP in the inferior leads and all-cause mortality among individuals aged 65-74 years. The hazard ratios and *P*-values were calculated by the Cox proportional hazards model after adjusting for covariables.

Variable		N	Hazard ratio	HR (95% CI)	p-Value
**ERP**	-	942		Reference	
	inf. +	121		0.86 (0.63, 1.19)	0.361
**Gender**	Male	552		Reference	
	Female	511		0.84 (0.63, 1.12)	0.242
**BMI**		1063		0.92 (0.89, 0.95)	<0.001
**Systolic_BP**		1063		1.02 (1.01, 1.03)	<0.001
**Diastolic_BP**		1063		0.98 (0.97, 0.99)	0.002
**Smoke**	Current	104		Reference	
	Quitted	245		0.77 (0.55, 1.08)	0.134
	Never	714		0.63 (0.44, 0.90)	0.011
**Hypertension**	0	467		Reference	
	1	596		0.85 (0.61, 1.18)	0.333
**Diabetes_Mellitus**	0	852		Reference	
	1	211		1.44 (1.13, 1.85)	0.004
**Stroke**	0	987		Reference	
	1	76		1.86 (1.32, 2.61)	<0.001
**Hyperlipidemia**	0	757		Reference	
	1	306		0.81 (0.63, 1.03)	0.086
**Chronic_Kidney_Disease**	0	940		Reference	
	1	123		1.23 (0.90, 1.68)	0.189
**QTcB**		1063		1.01 (1.00, 1.01)	0.014

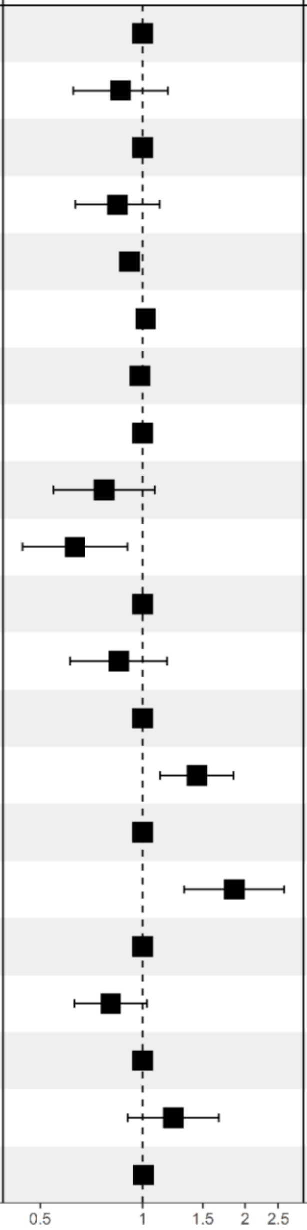

Supplementary Figure 17. Association between positive ERP in the inferior leads and all-cause mortality among individuals aged ≥75 years. The hazard ratios and *P*-values were calculated by the Cox proportional hazards model after adjusting for covariables.

Variable		N	HR (95% CI)	p-Value
ERP	-	1117	Reference	
	lat. +	95	1.89 (0.88, 4.08)	0.104
Gender	Male	568	Reference	
	Female	644	0.26 (0.11, 0.60)	0.002
BMI		1212	0.96 (0.88, 1.04)	0.282
Systolic_BP		1212	1.02 (0.99, 1.05)	0.255
Diastolic_BP		1212	0.97 (0.93, 1.01)	0.157
Smoke	Current	193	Reference	
	Quitted	151	0.59 (0.28, 1.22)	0.154
	Never	868	0.39 (0.19, 0.79)	0.009
Hypertension	0	839	Reference	
	1	373	0.99 (0.41, 2.39)	0.985
Diabetes_Mellitus	0	1068	Reference	
	1	144	2.07 (1.06, 4.08)	0.034
Stroke	0	1180	Reference	
	1	32	1.47 (0.44, 4.88)	0.529
Hyperlipidemia	0	817	Reference	
	1	395	0.71 (0.38, 1.34)	0.289
Chronic_Kidney_Disease	0	1011	Reference	
	1	201	1.55 (0.83, 2.89)	0.166
QTcB		1212	1.01 (1.00, 1.03)	0.022

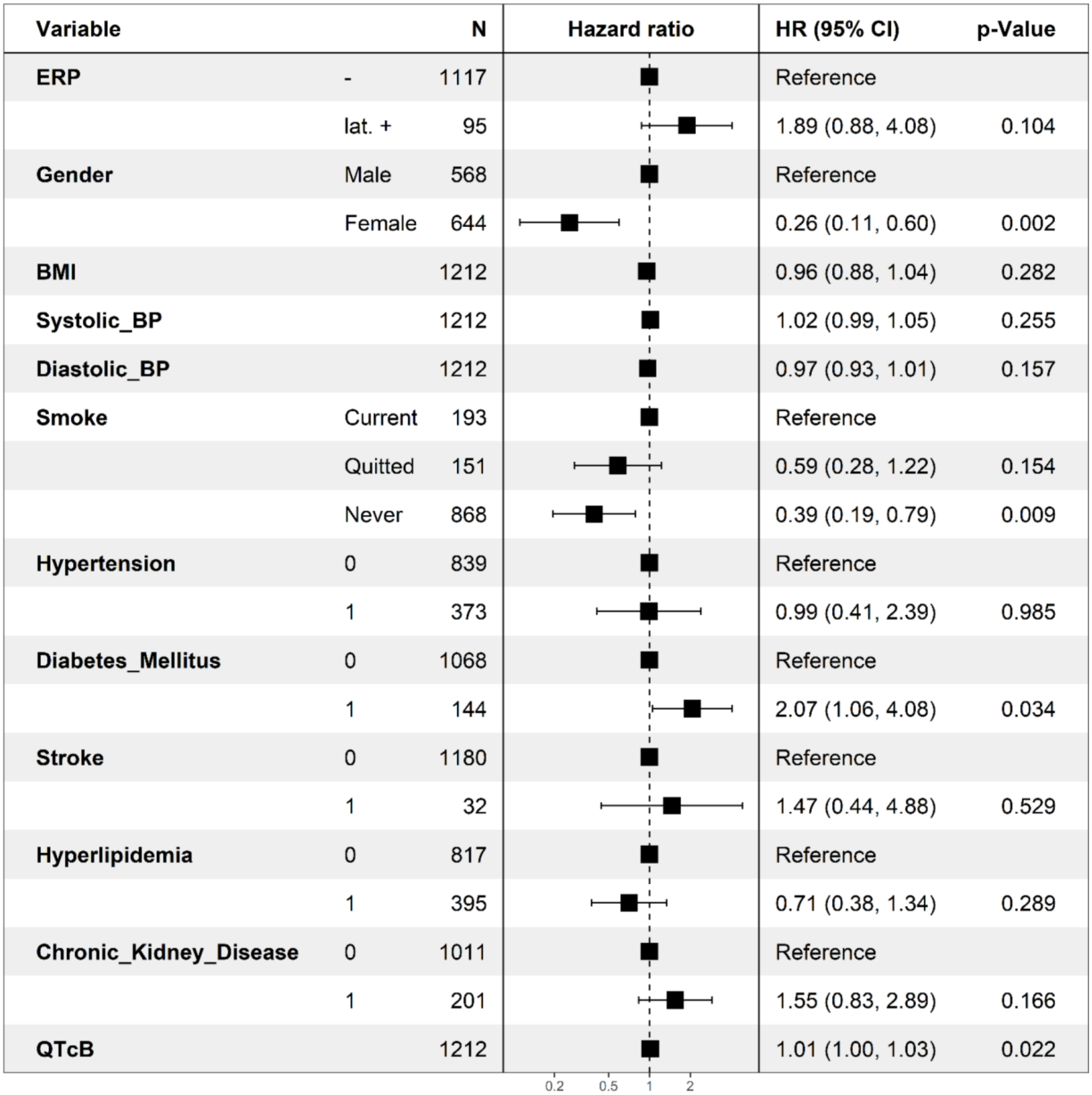

Supplementary Figure 18. Association between positive ERP in the lateral leads and all-cause mortality among individuals aged 55-64 years. The hazard ratios and *P*-values were calculated by the Cox proportional hazards model after adjusting for covariables.

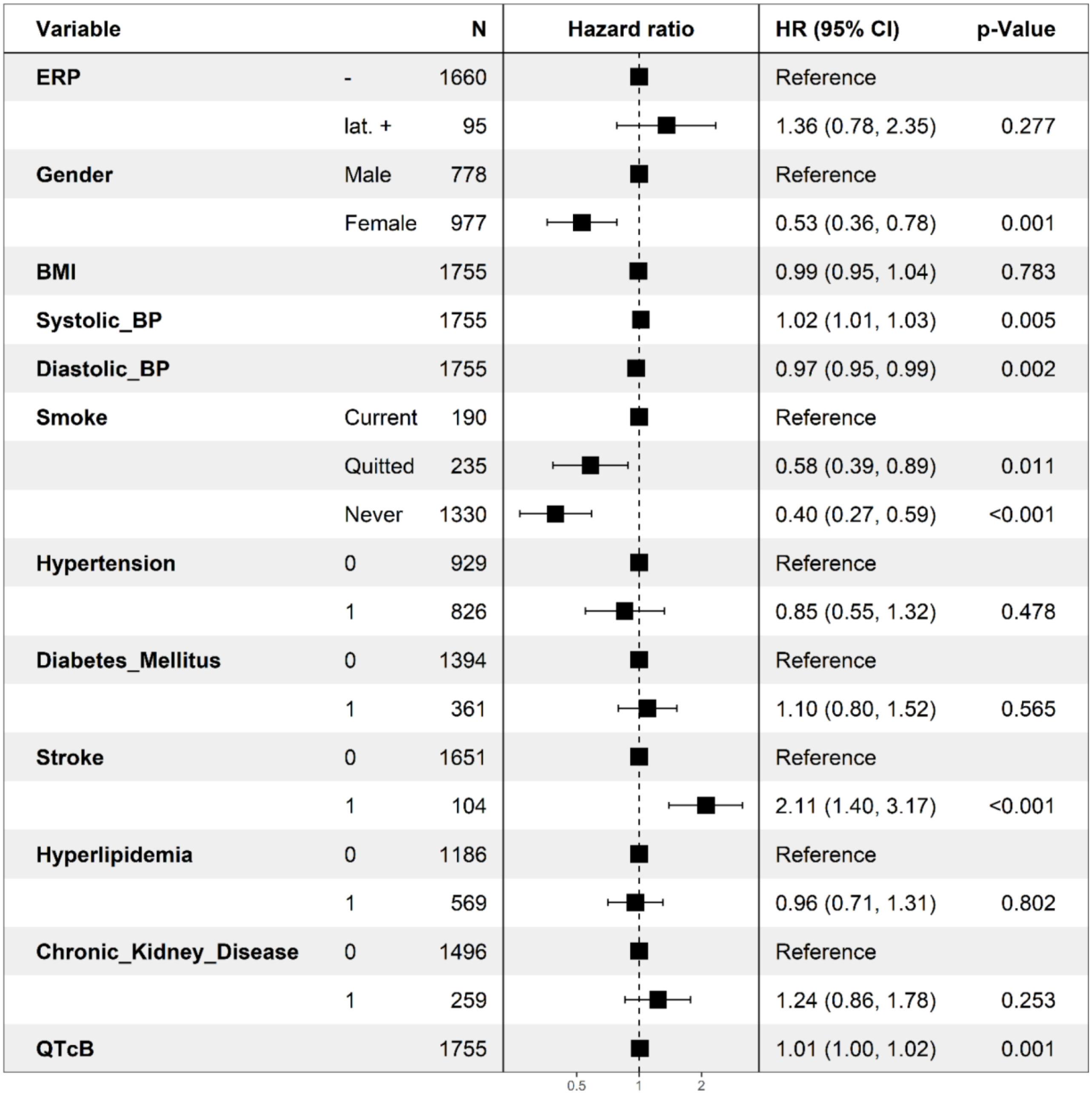

Supplementary Figure 19. Association between positive ERP in the lateral leads and all-cause mortality among individuals aged 65-74 years. The hazard ratios and *P*-values were calculated by the Cox proportional hazards model after adjusting for covariables.

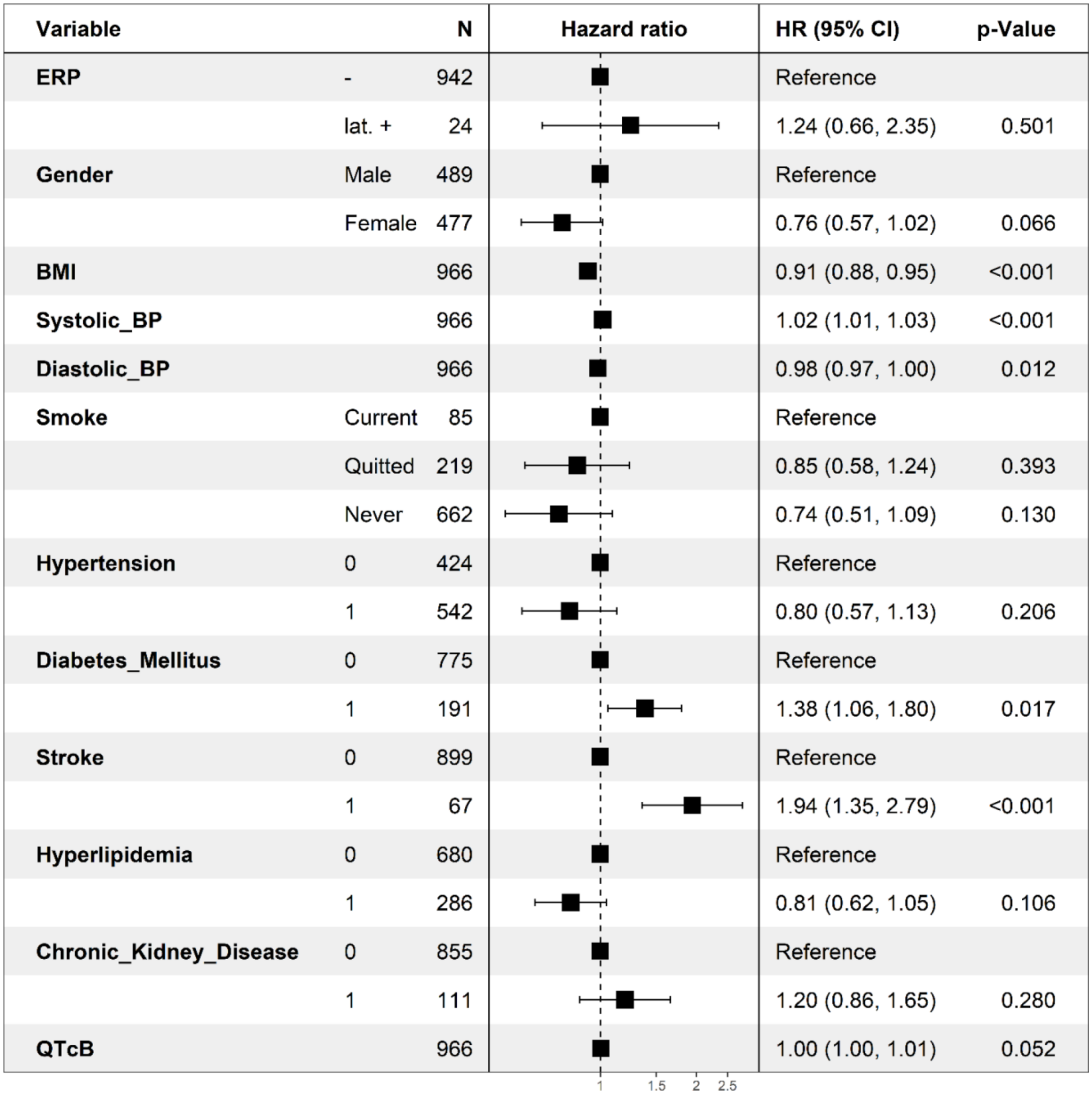

Supplementary Figure 20. Association between positive ERP in the lateral leads and all-cause mortality among those aged ≥75 years. The hazard ratios and *P*-values were calculated by the Cox proportional hazards model after adjusting for covariables.

Variable		N	Hazard ratio	HR (95% CI)	p-Value
**ERP**	-	1117		Reference	
	both +	43		0.48 (0.07, 3.55)	0.475
**Gender**	Male	543		Reference	
	Female	617		0.24 (0.10, 0.59)	0.002
**BMI**		1160		0.94 (0.87, 1.03)	0.185
**Systolic_BP**		1160		1.01 (0.98, 1.04)	0.437
**Diastolic_BP**		1160		0.99 (0.94, 1.03)	0.547
**Smoke**	Current	182		Reference	
	Quitted	144		0.57 (0.25, 1.30)	0.181
	Never	834		0.47 (0.22, 0.97)	0.041
**Hypertension**	0	807		Reference	
	1	353		0.95 (0.37, 2.44)	0.923
**Diabetes_Mellitus**	0	1023		Reference	
	1	137		2.11 (1.04, 4.30)	0.039
**Stroke**	0	1130		Reference	
	1	30		1.67 (0.50, 5.61)	0.404
**Hyperlipidemia**	0	783		Reference	
	1	377		0.75 (0.39, 1.47)	0.406
**Chronic_Kidney_Disease**	0	967		Reference	
	1	193		1.67 (0.87, 3.21)	0.123
**QTcB**		1160		1.01 (1.00, 1.03)	0.020

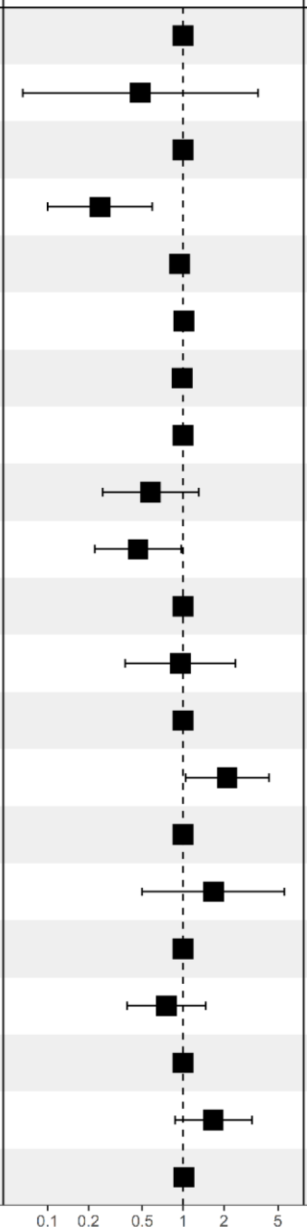

Supplementary Figure 21. Association between positive ERP in both inferior leads and lateral leads and all-cause mortality among individuals aged 55-64 years. The hazard ratios and *P*-values were calculated by the Cox proportional hazards model after adjusting for covariables.

Variable		N	HR (95% CI)	p-Value
**ERP**	-	1660	Reference	
	both +	46	0.47 (0.15, 1.48)	0.195
**Gender**	Male	767	Reference	
	Female	939	0.54 (0.36, 0.82)	0.003
**BMI**		1706	0.98 (0.94, 1.02)	0.400
**Systolic_BP**		1706	1.02 (1.01, 1.04)	0.002
**Diastolic_BP**		1706	0.97 (0.95, 0.99)	0.001
**Smoke**	Current	190	Reference	
	Quitted	240	0.60 (0.40, 0.91)	0.017
	Never	1276	0.38 (0.25, 0.58)	<0.001
**Hypertension**	0	902	Reference	
	1	804	0.85 (0.54, 1.33)	0.471
**Diabetes_Mellitus**	0	1352	Reference	
	1	354	1.12 (0.81, 1.56)	0.495
**Stroke**	0	1607	Reference	
	1	99	2.16 (1.42, 3.28)	<0.001
**Hyperlipidemia**	0	1149	Reference	
	1	557	1.07 (0.78, 1.45)	0.682
**Chronic_Kidney_Disease**	0	1455	Reference	
	1	251	1.27 (0.88, 1.83)	0.209
**QTcB**		1706	1.01 (1.00, 1.02)	0.001

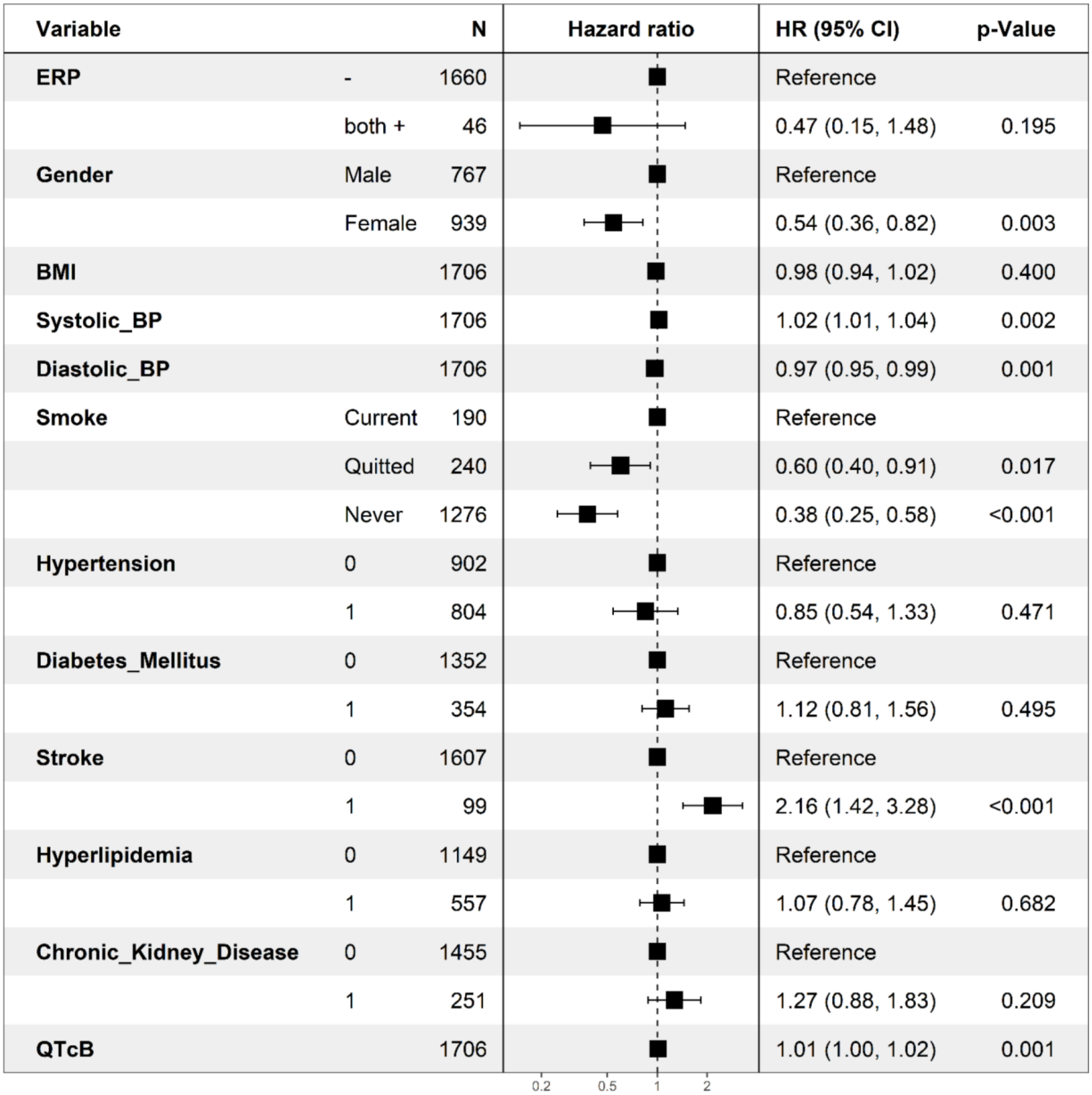

Supplementary Figure 22. Association between positive ERP in both inferior and lateral leads and all-cause mortality among individuals aged 65-74 years. The hazard ratios and *P*-values were calculated by the Cox proportional hazards model after adjusting for covariables.

Variable		N	HR (95% CI)	p-Value
**ERP**	-	942	Reference	
	both +	12	0.21 (0.03, 1.53)	0.124
**Gender**	Male	483	Reference	
	Female	471	0.79 (0.58, 1.06)	0.119
**BMI**		954	0.92 (0.89, 0.95)	<0.001
**Systolic_BP**		954	1.02 (1.01, 1.03)	<0.001
**Diastolic_BP**		954	0.98 (0.97, 1.00)	0.014
**Smoke**	Current	85	Reference	
	Quitted	218	0.85 (0.58, 1.24)	0.404
	Never	651	0.71 (0.48, 1.05)	0.083
**Hypertension**	0	415	Reference	
	1	539	0.80 (0.57, 1.13)	0.213
**Diabetes_Mellitus**	0	768	Reference	
	1	186	1.42 (1.09, 1.86)	0.010
**Stroke**	0	888	Reference	
	1	66	1.90 (1.32, 2.75)	0.001
**Hyperlipidemia**	0	673	Reference	
	1	281	0.78 (0.60, 1.02)	0.070
**Chronic_Kidney_Disease**	0	845	Reference	
	1	109	1.20 (0.86, 1.67)	0.278
**QTcB**		954	1.00 (1.00, 1.01)	0.072

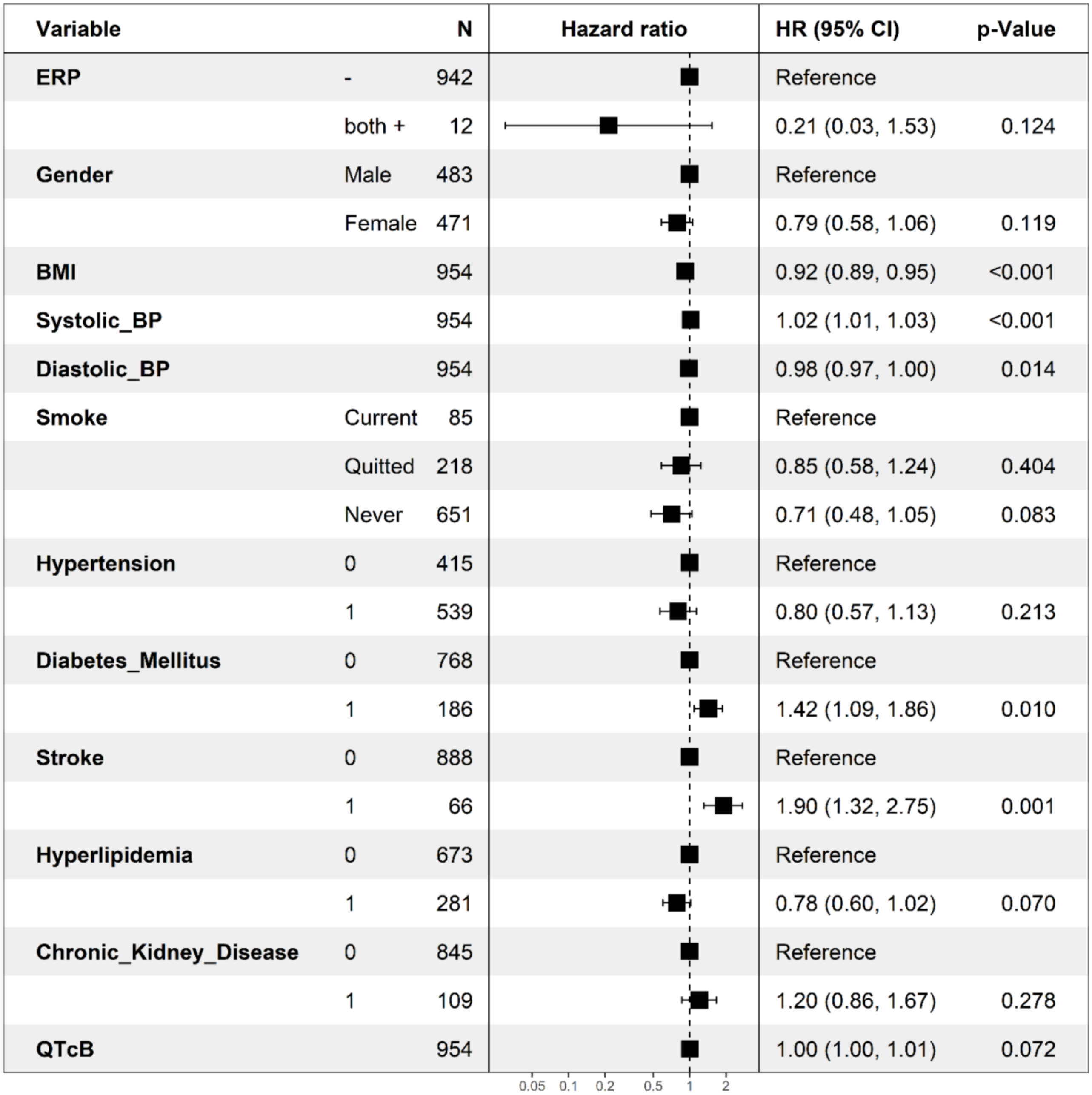

Supplementary Figure 23. Association between positive ERP in both inferior and lateral leads and all-cause mortality among individuals aged ≥75 years. The hazard ratios and *P*-values were calculated by the Cox proportional hazards model after adjusting for covariables.

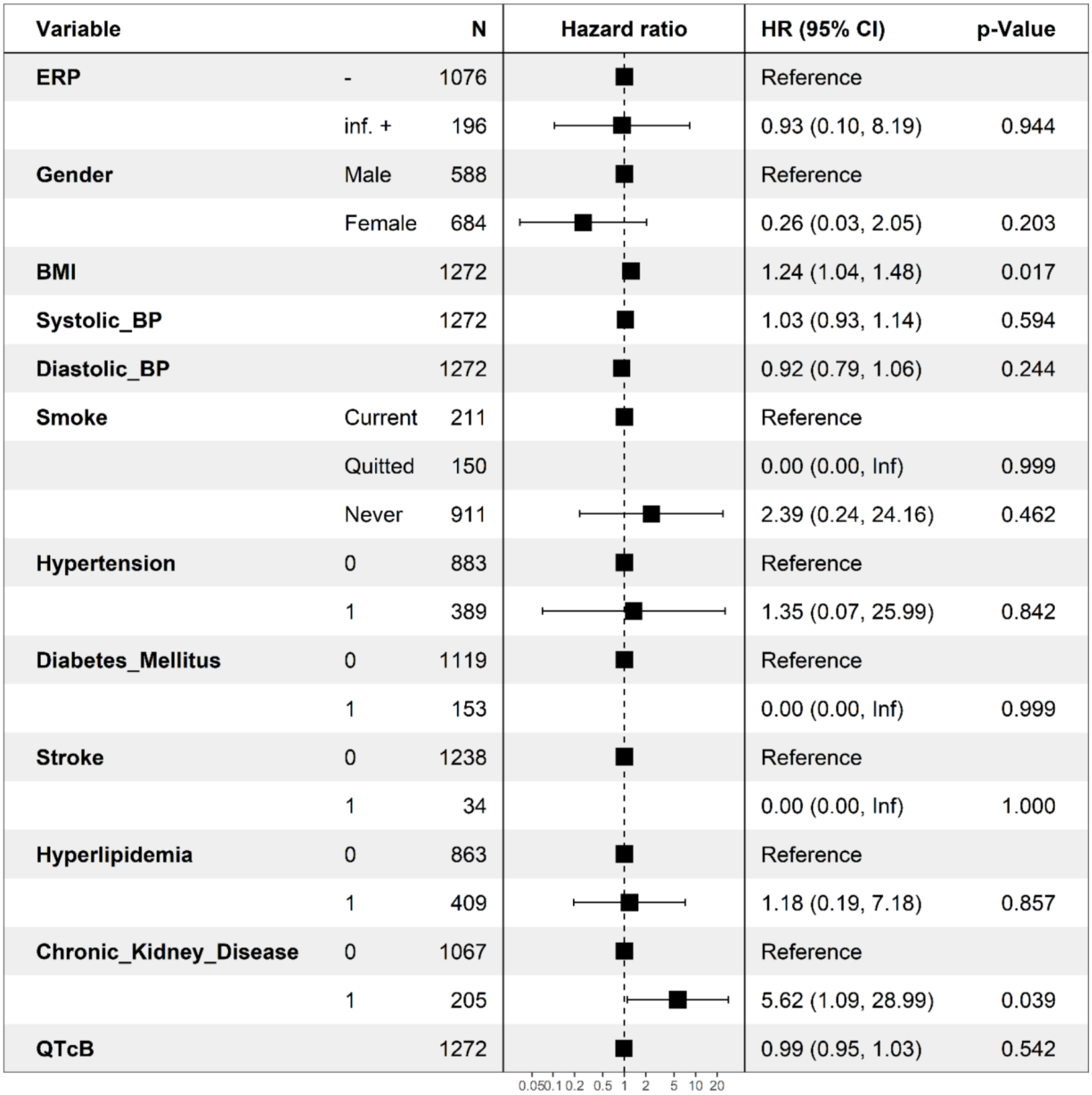

Supplementary Figure 24. Association between positive ERP in the inferior leads and cardiovascular mortality among individuals aged 55-64 years. The hazard ratios and *P*-values were calculated by the Cox proportional hazards model after adjusting for covariables.

Variable		N	HR (95% CI)	p-Value
**ERP**	-	1489	Reference	
	inf. +	221	0.93 (0.27, 3.21)	0.913
**Gender**	Male	733	Reference	
	Female	977	0.30 (0.10, 0.90)	0.032
**BMI**		1710	1.02 (0.91, 1.15)	0.743
**Systolic_BP**		1710	1.00 (0.97, 1.04)	0.822
**Diastolic_BP**		1710	1.00 (0.95, 1.05)	0.963
**Smoke**	Current	167	Reference	
	Quitted	229	0.90 (0.26, 3.04)	0.861
	Never	1314	0.74 (0.23, 2.45)	0.626
**Hypertension**	0	919	Reference	
	1	791	1.62 (0.47, 5.63)	0.449
**Diabetes_Mellitus**	0	1368	Reference	
	1	342	1.22 (0.49, 3.06)	0.672
**Stroke**	0	1627	Reference	
	1	83	3.57 (1.29, 9.87)	0.014
**Hyperlipidemia**	0	1138	Reference	
	1	572	0.90 (0.38, 2.13)	0.805
**Chronic_Kidney_Disease**	0	1467	Reference	
	1	243	0.26 (0.04, 1.96)	0.192
**QTcB**		1710	1.02 (1.01, 1.04)	0.006

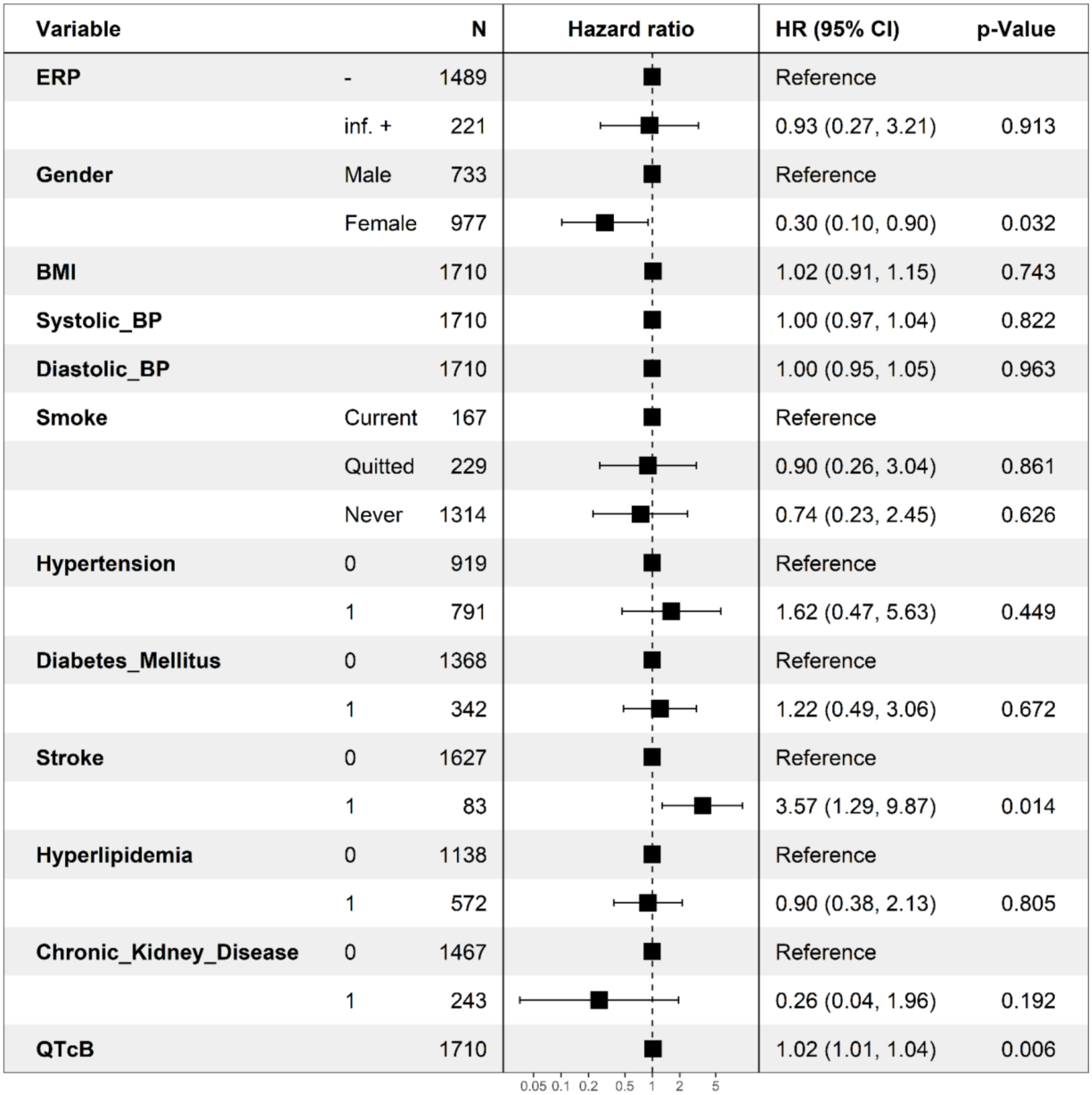

Supplementary Figure 25. Association between positive ERP in the inferior leads and cardiovascular mortality among individuals aged 65-74 years. The hazard ratios and *P*-values were calculated by the Cox proportional hazards model after adjusting for covariables.

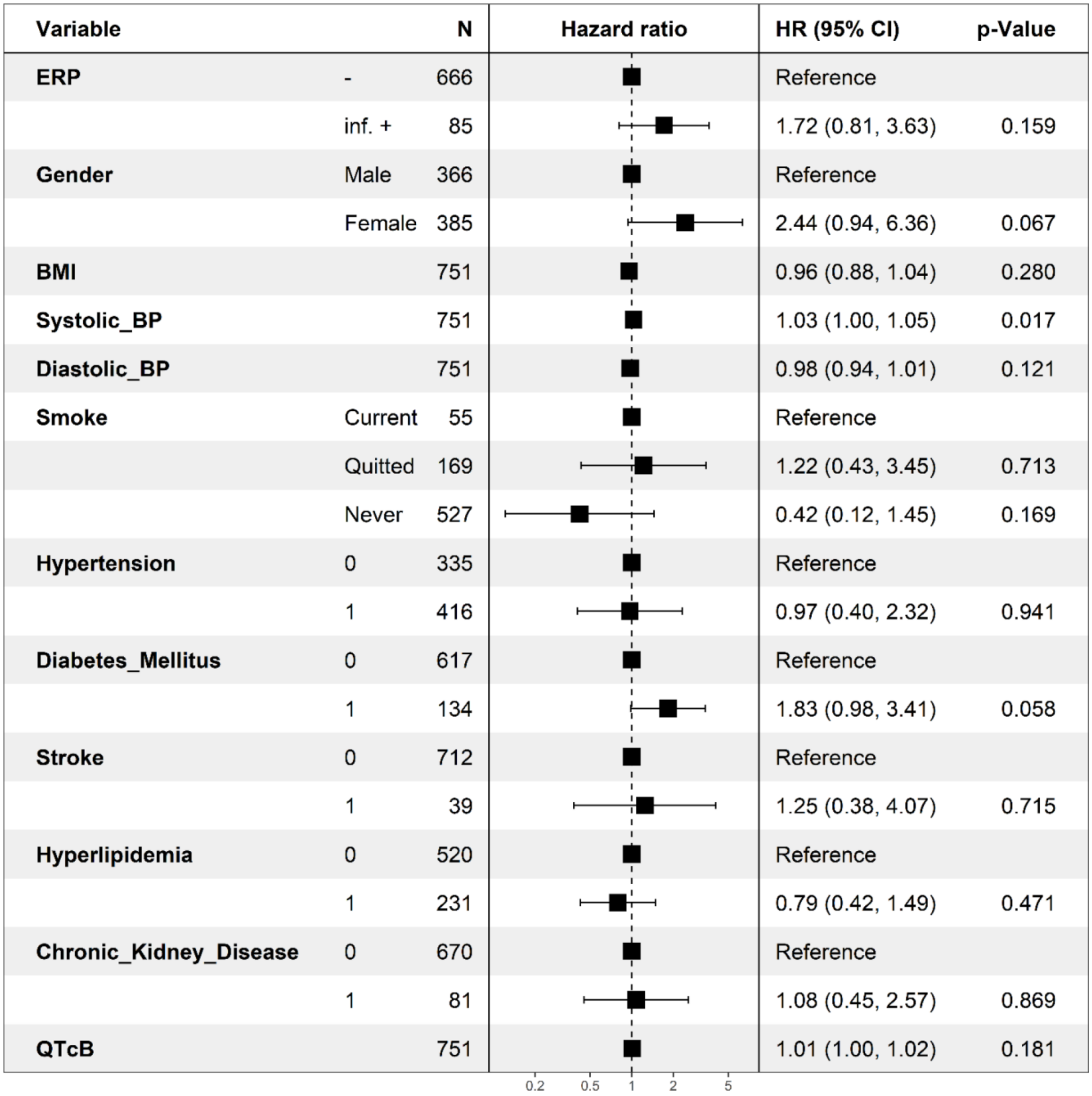

Supplementary Figure 26. Association between positive ERP in the inferior leads and cardiovascular mortality among individuals aged ≥75 years. The hazard ratios and *P*-values were calculated by the Cox proportional hazards model after adjusting for covariables.

Variable		N	HR (95% CI)	p-Value
**ERP**	-	1076	Reference	
	lat. +	88	1.99 (0.21, 18.98)	0.549
**Gender**	Male	529	Reference	
	Female	635	0.18 (0.02, 2.06)	0.166
**BMI**		1164	1.27 (1.03, 1.58)	0.029
**Systolic_BP**		1164	1.01 (0.91, 1.12)	0.865
**Diastolic_BP**		1164	0.91 (0.79, 1.05)	0.189
**Smoke**	Current	173	Reference	
	Quitted	140	0.00 (0.00, Inf)	0.999
	Never	851	1.29 (0.20, 8.23)	0.788
**Hypertension**	0	809	Reference	
	1	355	2.42 (0.10, 60.13)	0.590
**Diabetes_Mellitus**	0	1033	Reference	
	1	131	0.00 (0.00, Inf)	0.999
**Stroke**	0	1135	Reference	
	1	29	0.00 (0.00, Inf)	1.000
**Hyperlipidemia**	0	782	Reference	
	1	382	0.48 (0.05, 4.70)	0.528
**Chronic_Kidney_Disease**	0	974	Reference	
	1	190	6.19 (1.17, 32.85)	0.032
**QTcB**		1164	0.98 (0.94, 1.02)	0.317

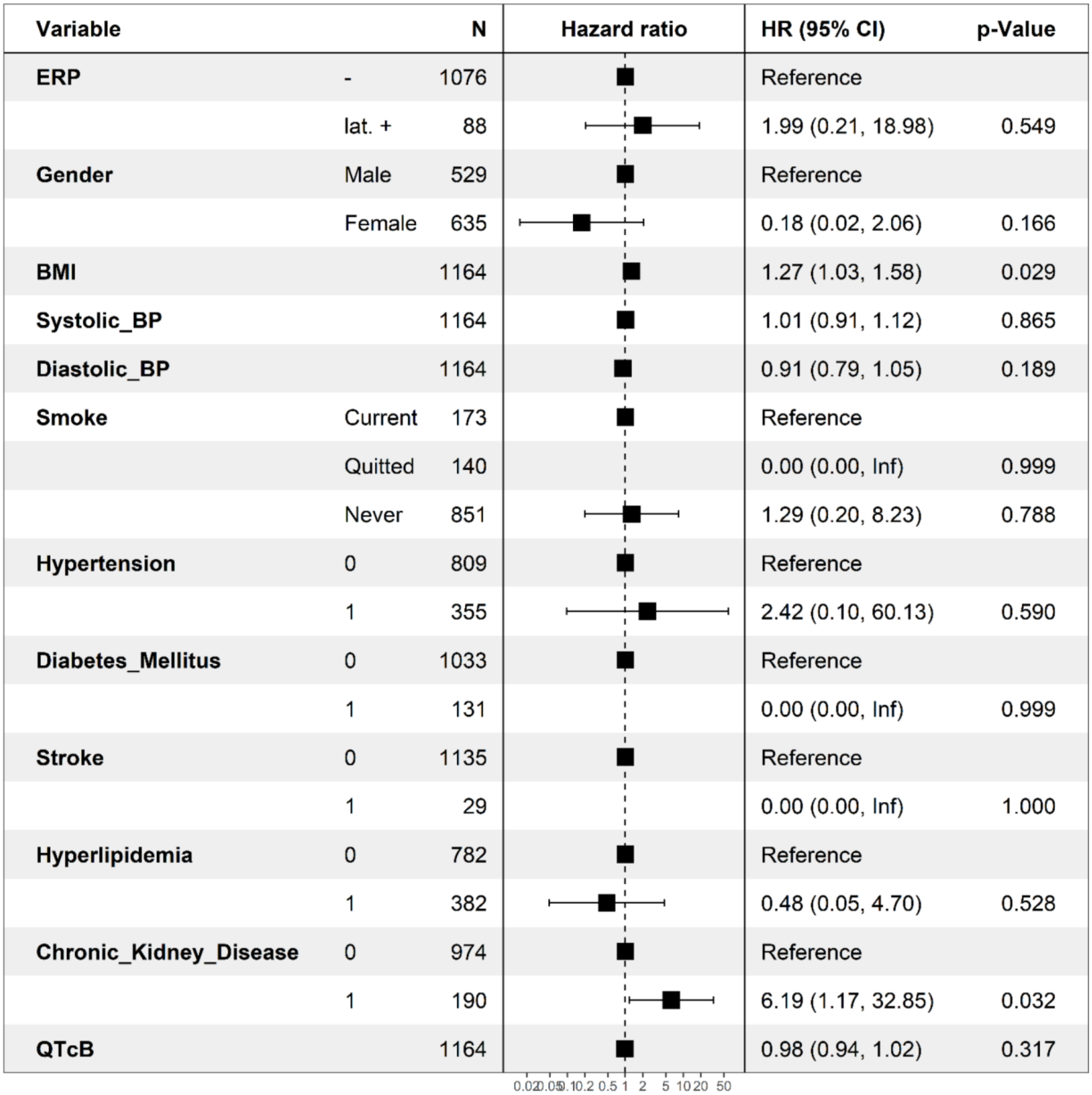

Supplementary Figure 27. Association between positive ERP in the lateral leads and cardiovascular mortality among individuals aged 55-64 years. The hazard ratios and *P*-values were calculated by the Cox proportional hazards model after adjusting for covariables.

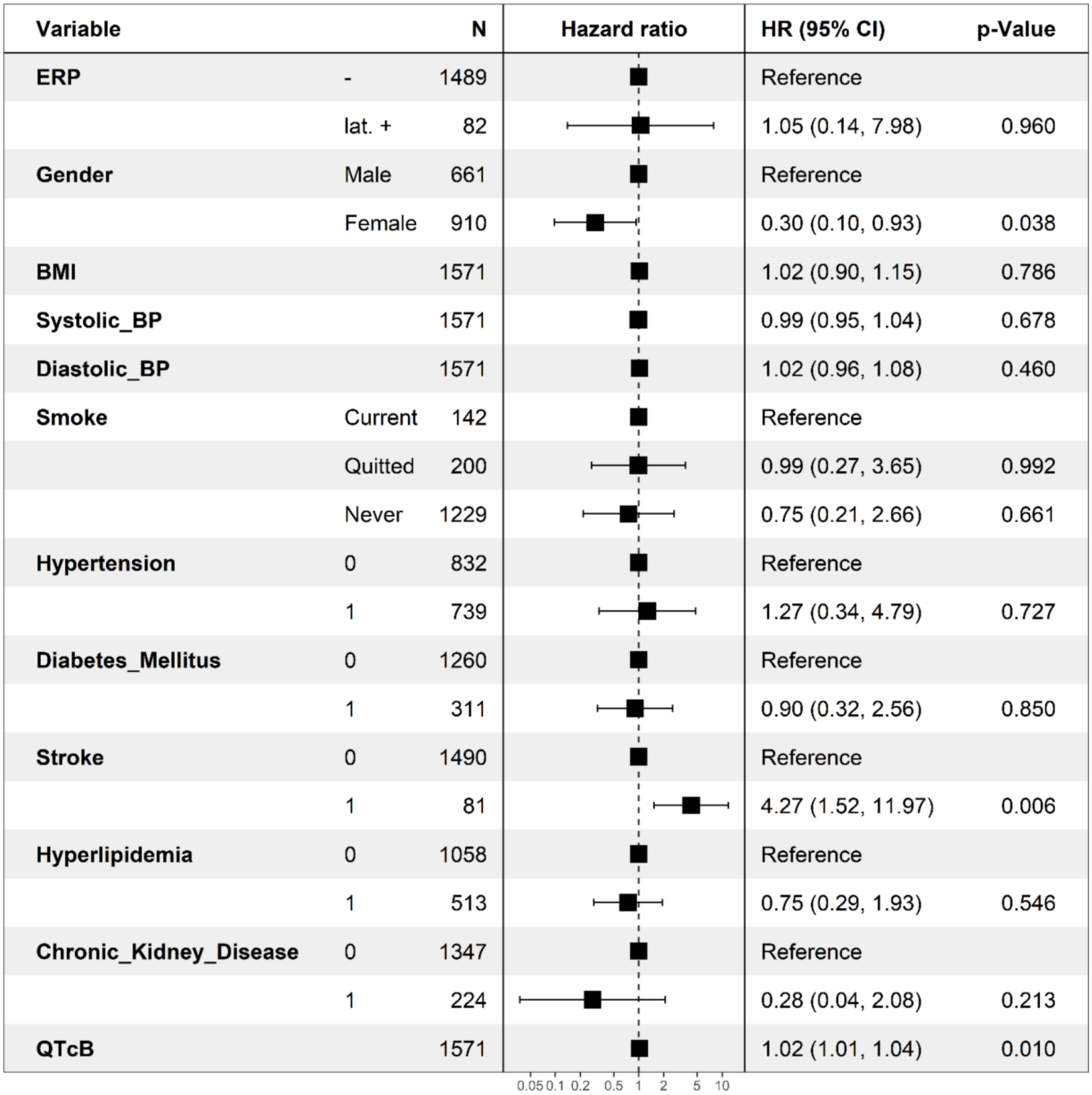

Variable		N	HR (95% CI)	p-Value
**ERP**	-	1489	Reference	
	lat. +	82	1.05 (0.14, 7.98)	0.960
**Gender**	Male	661	Reference	
	Female	910	0.30 (0.10, 0.93)	0.038
**BMI**		1571	1.02 (0.90, 1.15)	0.786
**Systolic_BP**		1571	0.99 (0.95, 1.04)	0.678
**Diastolic_BP**		1571	1.02 (0.96, 1.08)	0.460
**Smoke**	Current	142	Reference	
	Quitted	200	0.99 (0.27, 3.65)	0.992
	Never	1229	0.75 (0.21, 2.66)	0.661
**Hypertension**	0	832	Reference	
	1	739	1.27 (0.34, 4.79)	0.727
**Diabetes_Mellitus**	0	1260	Reference	
	1	311	0.90 (0.32, 2.56)	0.850
**Stroke**	0	1490	Reference	
	1	81	4.27 (1.52, 11.97)	0.006
**Hyperlipidemia**	0	1058	Reference	
	1	513	0.75 (0.29, 1.93)	0.546
**Chronic_Kidney_Disease**	0	1347	Reference	
	1	224	0.28 (0.04, 2.08)	0.213
**QTcB**		1571	1.02 (1.01, 1.04)	0.010

Supplementary Figure 28. Association between positive ERP in the lateral leads and cardiovascular mortality among individuals aged 65-74 years. The hazard ratios and *P*-values were calculated by the Cox proportional hazards model after adjusting for covariables.

Variable		N	HR (95% CI)	p-Value
**ERP**	-	666	Reference	
	lat. +	16	1.99 (0.47, 8.37)	0.348
**Gender**	Male	320	Reference	
	Female	362	1.93 (0.72, 5.16)	0.191
**BMI**		682	0.93 (0.85, 1.01)	0.092
**Systolic_BP**		682	1.04 (1.01, 1.06)	0.002
**Diastolic_BP**		682	0.98 (0.95, 1.01)	0.220
**Smoke**	Current	46	Reference	
	Quitted	150	2.63 (0.58, 11.87)	0.210
	Never	486	1.01 (0.19, 5.35)	0.989
**Hypertension**	0	299	Reference	
	1	383	0.63 (0.25, 1.60)	0.329
**Diabetes_Mellitus**	0	560	Reference	
	1	122	1.57 (0.78, 3.14)	0.206
**Stroke**	0	647	Reference	
	1	35	1.69 (0.51, 5.59)	0.392
**Hyperlipidemia**	0	466	Reference	
	1	216	0.76 (0.38, 1.50)	0.424
**Chronic_Kidney_Disease**	0	610	Reference	
	1	72	1.11 (0.43, 2.87)	0.837
**QTcB**		682	1.00 (0.99, 1.02)	0.535

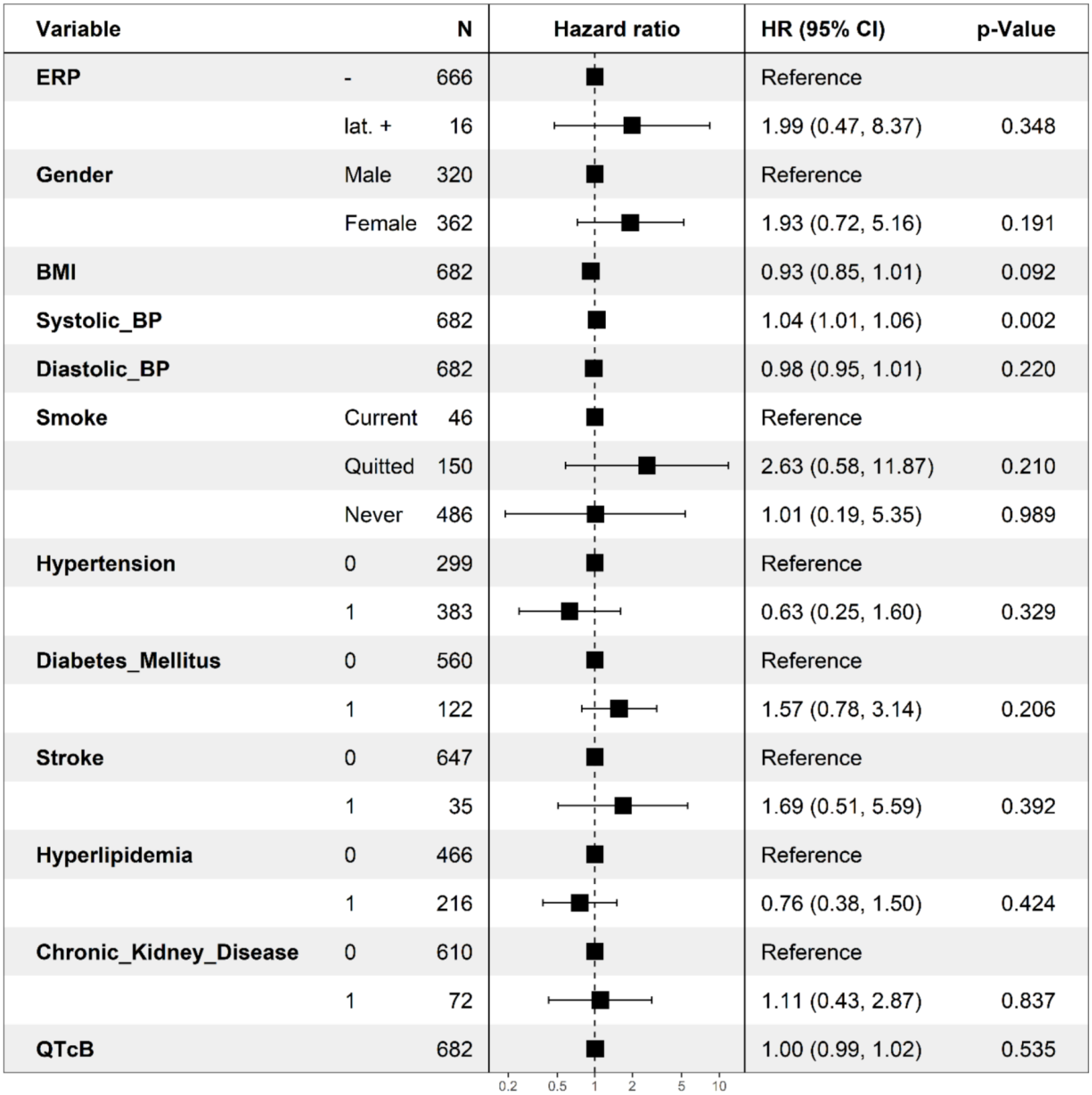

Supplementary Figure 29. Associations between positive ERP in the lateral leads and cardiovascular mortality among individuals aged ≥75 years. The hazard ratios and *P*-values were calculated by the Cox proportional hazards model after adjusting for covariables.

Variable		N	Hazard ratio	HR (95% CI)	p-Value
ERP	-	1076		Reference	
	both +	42		0.00 (0.00, Inf)	1.000
Gender	Male	509		Reference	
	Female	609		0.20 (0.02, 2.31)	0.196
BMI		1118		1.30 (1.05, 1.60)	0.017
Systolic_BP		1118		1.01 (0.90, 1.13)	0.836
Diastolic_BP		1118		0.93 (0.78, 1.09)	0.363
Smoke	Current	165		Reference	
	Quitted	135		0.00 (0.00, Inf)	0.999
	Never	818		2.87 (0.27, 30.91)	0.384
Hypertension	0	781		Reference	
	1	337		2.49 (0.08, 74.50)	0.598
Diabetes_Mellitus	0	993		Reference	
	1	125		0.00 (0.00, Inf)	0.999
Stroke	0	1091		Reference	
	1	27		0.00 (0.00, Inf)	1.000
Hyperlipidemia	0	753		Reference	
	1	365		0.44 (0.04, 5.58)	0.530
Chronic_Kidney_Disease	0	935		Reference	
	1	183		8.56 (1.32, 55.47)	0.024
QTcB		1118		0.98 (0.93, 1.02)	0.316

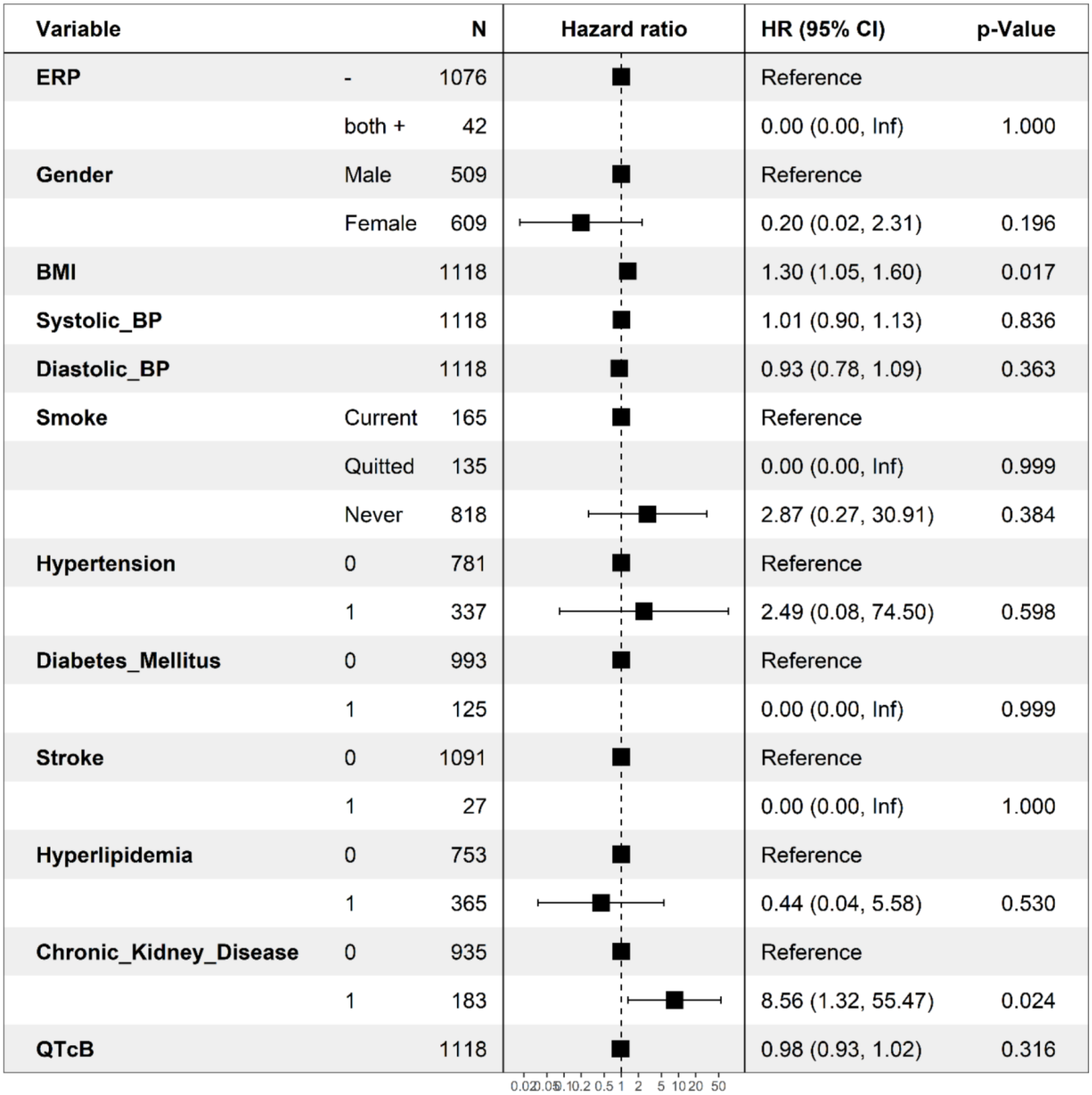

Supplementary Figure 30. Association between positive ERP in both inferior and lateral leads and cardiovascular mortality among individuals aged 55-64 years. The hazard ratios and *P*-values were calculated by the Cox proportional hazards model after adjusting for covariables.

Variable		N	HR (95% CI)	p-Value
**ERP**	-	1489	Reference	
	both +	43	0.00 (0.00, Inf)	0.998
**Gender**	Male	656	Reference	
	Female	876	0.23 (0.07, 0.76)	0.016
**BMI**		1532	1.02 (0.90, 1.16)	0.767
**Systolic_BP**		1532	1.00 (0.96, 1.04)	0.941
**Diastolic_BP**		1532	1.02 (0.97, 1.08)	0.440
**Smoke**	Current	144	Reference	
	Quitted	204	0.97 (0.26, 3.56)	0.962
	Never	1184	0.80 (0.23, 2.85)	0.732
**Hypertension**	0	811	Reference	
	1	721	1.11 (0.29, 4.21)	0.878
**Diabetes_Mellitus**	0	1227	Reference	
	1	305	0.95 (0.33, 2.68)	0.915
**Stroke**	0	1455	Reference	
	1	77	4.47 (1.59, 12.57)	0.005
**Hyperlipidemia**	0	1033	Reference	
	1	499	0.79 (0.30, 2.08)	0.637
**Chronic_Kidney_Disease**	0	1315	Reference	
	1	217	0.29 (0.04, 2.15)	0.224
**QTcB**		1532	1.03 (1.01, 1.05)	0.003

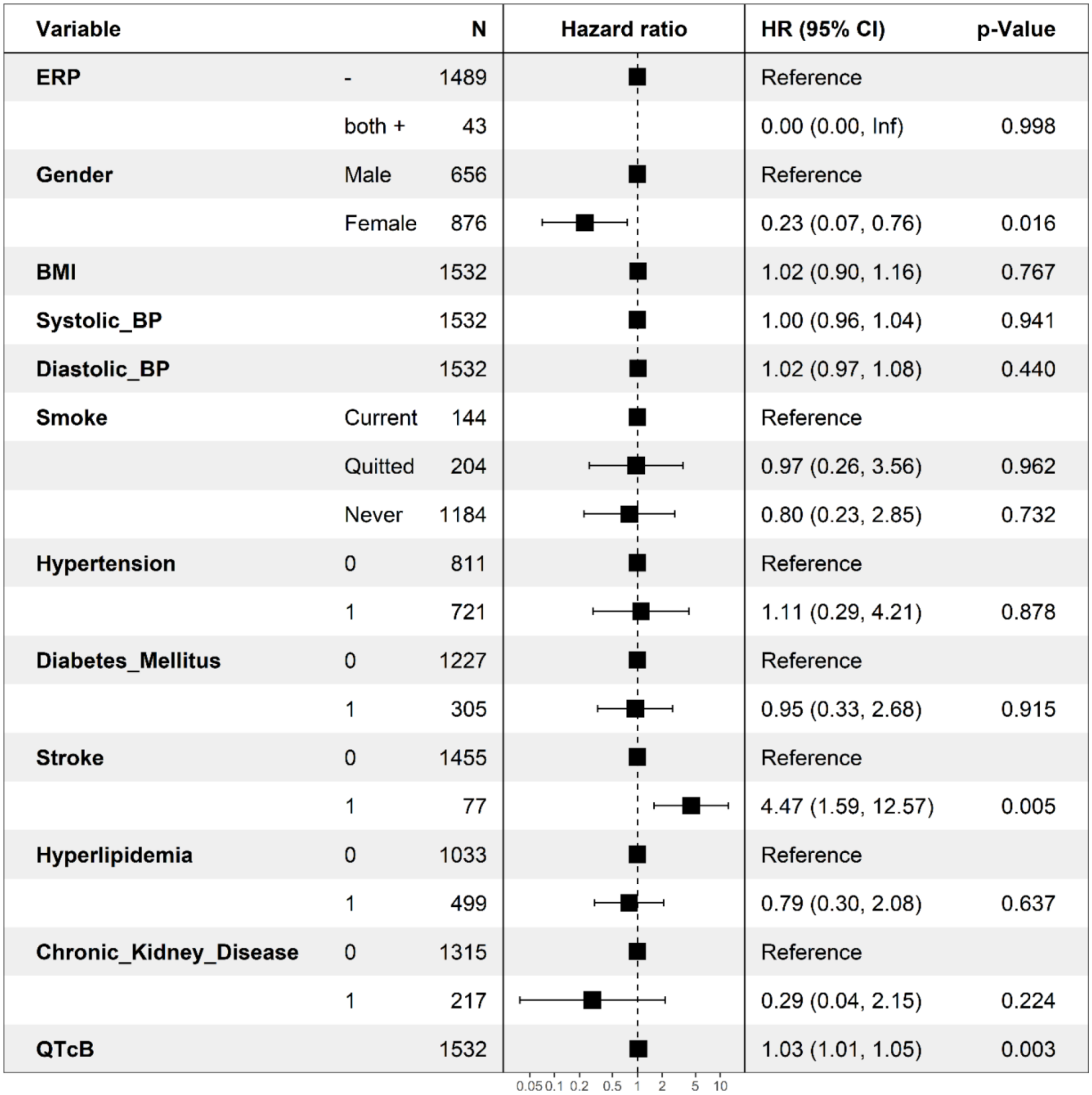

Supplementary Figure 31. Association between positive ERP in both inferior and lateral leads and cardiovascular mortality among individuals aged 65-74 years. The hazard ratios and *P*-values were calculated by the Cox proportional hazards model after adjusting for covariables.

Variable		N	HR (95% CI)	p-Value
**ERP**	-	666	Reference	
	both +	11	0.00 (0.00, Inf)	0.997
**Gender**	Male	318	Reference	
	Female	359	2.30 (0.82, 6.49)	0.115
**BMI**		677	0.94 (0.85, 1.03)	0.163
**Systolic_BP**		677	1.04 (1.01, 1.06)	0.002
**Diastolic_BP**		677	0.98 (0.94, 1.01)	0.189
**Smoke**	Current	46	Reference	
	Quitted	150	2.67 (0.59, 12.13)	0.205
	Never	481	0.81 (0.15, 4.44)	0.811
**Hypertension**	0	294	Reference	
	1	383	0.69 (0.27, 1.80)	0.451
**Diabetes_Mellitus**	0	558	Reference	
	1	119	1.64 (0.81, 3.31)	0.168
**Stroke**	0	642	Reference	
	1	35	1.65 (0.50, 5.48)	0.412
**Hyperlipidemia**	0	463	Reference	
	1	214	0.78 (0.39, 1.56)	0.485
**Chronic_Kidney_Disease**	0	605	Reference	
	1	72	1.18 (0.45, 3.07)	0.739
**QTcB**		677	1.00 (0.99, 1.02)	0.505

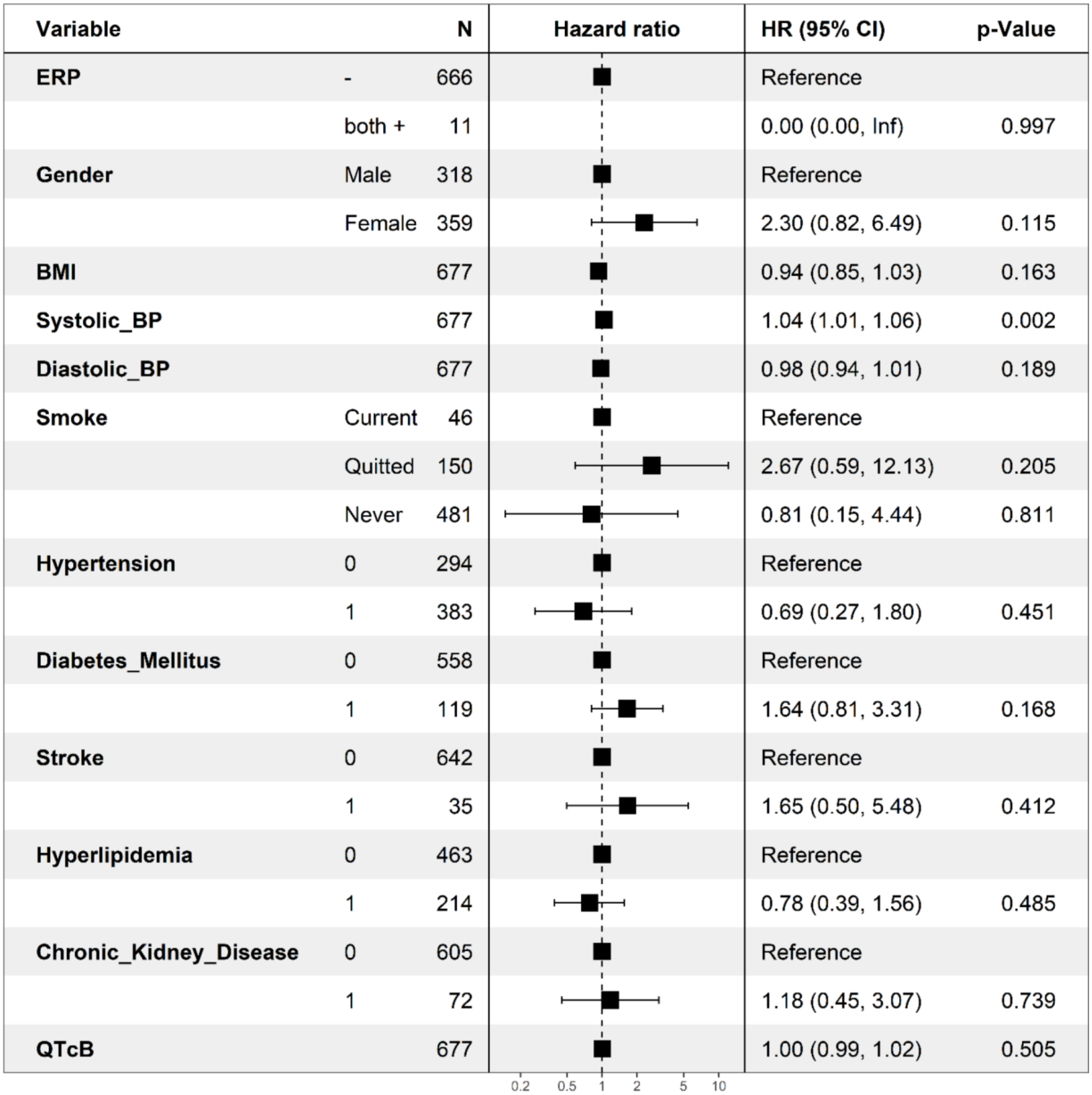

Supplementary Figure 32. Association between positive ERP in both inferior and lateral leads and cardiovascular mortality among those aged ≥75 years. The hazard ratios and *P*-values were calculated by the Cox proportional hazards model after adjusting for covariables.
